# The GET pathway is a major bottleneck for maintaining proteostasis in *Saccharomyces cerevisiae*

**DOI:** 10.1038/s41598-023-35666-8

**Published:** 2023-06-07

**Authors:** Rebecca Josefson, Navinder Kumar, Xinxin Hao, Beidong Liu, Thomas Nyström

**Affiliations:** 1 Department of Microbiology and Immunology, Institute of Biomedicine, Sahlgrenska Academy, University of Gothenburg, Gothenburg, Sweden; 2 Department of Chemistry and Molecular Biology, Faculty of Science, University of Gothenburg, Gothenburg, Sweden

**Keywords:** Protein aggregation, Protein quality control

## Abstract

A hallmark of aging in a variety of organisms is a breakdown of proteostasis and an ensuing accumulation of protein aggregates and inclusions. However, it is not clear if the proteostasis network suffers from a uniform breakdown during aging or if some distinct components act as bottlenecks especially sensitive to functional decline. Here, we report on a genome-wide, unbiased, screen for single genes in young cells of budding yeast required to keep the proteome aggregate-free under non-stress conditions as a means to identify potential proteostasis bottlenecks. We found that the GET pathway, required for the insertion of tail-anchored (TA) membrane proteins in the endoplasmic reticulum, is such a bottleneck as single mutations in either *GET3*, *GET2* or *GET1* caused accumulation of cytosolic Hsp104- and mitochondria-associated aggregates in nearly all cells when growing at 30 °C (non-stress condition). Further, results generated by a second screen identifying proteins aggregating in GET mutants and analyzing the behavior of cytosolic reporters of misfolding, suggest that there is a general collapse in proteostasis in GET mutants that affects other proteins than TA proteins.

## Introduction

The proteostasis network^1,2^ is dedicated to the folding of newly synthesized proteins, refolding of misfolded proteins, and promoting degradation of irreparable and/or superfluous proteins. When this network becomes overwhelmed, as during a heat shock, misfolded proteins accumulate and form aggregates. Additionally, accumulation of damaged, misfolded proteins, and aggregated proteins is one of the hallmarks of aging^3,4^. Aggregates can cause fitness defects as the function of the native protein is lost, by formation of toxic non-native protein–protein interactions, and by depletion of proteostasis components^5,6^. The proteostasis network is commonly used to describe systems directly involved in protein folding and stability, i.e. translation components, chaperones, cochaperones and the ubiquitin–proteasome (UPS) and autophagy systems, in addition to connected signaling pathways and modifiers (e.g. stress responses and nutrient signaling) affecting proteostasis capacity^2,7^. However, it is clear that many auxiliary factors not typically associated with the canonical factors of the proteostasis network are key factors in keeping the proteome functional and intact^8–10^.

Several gene deletions have been found to cause increased protein aggregation in yeast cells cultivated under non-stress conditions (e.g. steady-state growth in rich medium at 30 °C). For example, mutations causing defects in protein degradation results in the accumulation of several reporters of misfolded and aggregated proteins, such as CPY and Ste6^11,12^. Mutations in genes of the nascent polypeptide-associated complex (NAC) and the ribosome-associated Hsp70s, Ssb1 and Ssb2, likewise cause accumulation of aggregated proteins^13^. Additionally, a double mutant lacking the cytosolic Hsp70s genes, *SSA1* and *SSA2*, accumulates aggregates of several fluorescent, misfolding reporters^14,15^ and a deletion of the yeast thioredoxin gene, *TSA1*, which can act as a cochaperone during oxidative stress, causes the accumulation of aggregated ribosomal proteins^16,17^ during non-stress. However, genome-wide screens suggest that the canonical proteostasis genes, i.e. those encoding chaperones and co-chaperones are genetically well-buffered. Such screens have instead pinpointed auxiliary factors, not obviously connected to protein quality control, as key limiting components of the proteostasis network. Such factors include those involved in vesicle trafficking, polarity, actin cable functionality, and endocytosis^8–10,14,18,19^.

So far, screens have reported on mutations affecting proteostasis when protein quality control has been challenged. Here, we report on a genome-wide screen to identify genes that are required to keep the proteome aggregation-free during steady state growth under non-stress conditions. Finding such bottlenecks in proteostasis might help pinpoint weak and poorly buffered nodes of the proteostasis network. Using this approach, we identified the GET pathway as one of very few functions keeping cells aggregate-free and that reducing GET activity caused a general collapse in proteostasis and an increased sensitivity to neurological disease proteins.

## Results

### The GET pathway is a bottleneck in protein folding

**Figure 1:**
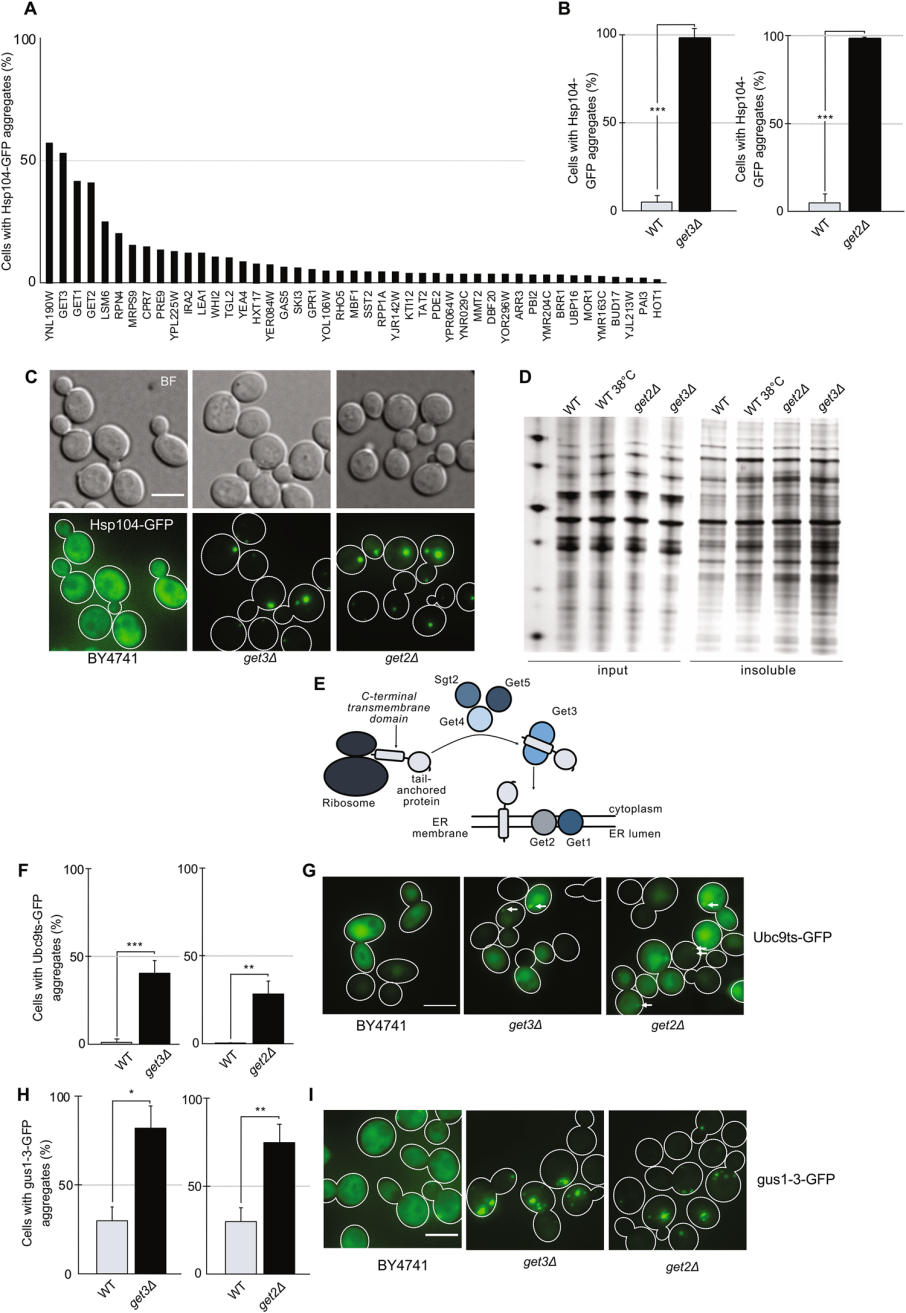
The GET pathway is a major bottleneck in protein folding. (**A**) Percentage of cells displaying Hsp104-GFP aggregates in the strains identified as hits in a screen for genes preventing protein aggregation in young, unstressed cells. The graph is based on quantification of the screen using high content microscopy. (**B**) Bar graphs show the percentage of mother cells with ≥ 1 Hsp104-GFP foci in *get3Δ* and *get2Δ* cells after growth to midlog phase at 30 °C (N = 3, n > 200 cells per strain per replicate). (**C**) Representative DIC and GFP images of Hsp104-GFP foci in wild type, *get3Δ*, and *get2Δ* cells. GFP channel images displayed as maximal Z projection (**D**) The insoluble protein fraction isolated from whole cell lysates of BY4741 wild type cells, BY4741 wild type cells heat shocked for 60 min at 38 °C, and *get3Δ* and *get2Δ* cells (N = 3). For full gel image, see Supplementary Fig. S2A, S2B. (**E**) Overview of the GET pathway for ER localization of TA proteins. (**F**) Ubc9ts-GFP foci in *get3Δ* cells at 30 °C after 5 h induction with galactose. Bar graphs show the percentage of mother cells with Ubc9ts-GFP foci (N = 3, n > 200 cells per strain per replicate). (**G**) Representative microscopy images of Ubc9ts-GFP foci in BY4741, *get3Δ* and *get2Δ* cells. White outlines show mother cells and daughter cells. (**H**) Aggregation of gus1-3-GFP in *get3Δ* and *get2Δ* cells. Bar graphs show the percentage of mother cells with gus1-3-GFP foci (N = 3, n > 200 cells per strain per replicate). (**I**) Representative images of *gus1-3-GFP* in BY4741, *get3Δ* and *get2Δ* cells. GFP channel displayed as maximal Z projection * p < 0.05, ** p < 0.01, *** p < 0.001, n.s. > 0.05, unpaired two-tailed t test. Scale bar 5 µm.

To find bottlenecks in protein folding, a genome-wide screen was performed to identify yeast deletion strains that accumulate protein aggregates during non-stress conditions (Supplementary Fig. S1A online). The Hsp104-GFP reporter was previously introduced into the yeast knockout library^8^. This library was grown to mid-exponential phase at 30 °C and mutant cells displaying Hsp104-GFP aggregates were identified using high-content microscopy (Fig. 1A; Supplemental table S1 online). Three out of the four top hits were proteins involved in the GET pathway; *GET3*, *GET2* and *GET1*. Indeed, manual verification of the screening results using a different genetic background (BY4741) showed that almost 100% of the cells carrying deletions in *GET3* (*YDL100C*), *GET2* (*YER083C*; Fig. 1B,C), and *GET1* (*YGL020C*; Supplementary Fig. S1B, S1C online), displayed Hsp104-GFP foci. Isolation of the insoluble fraction of proteins from *get2Δ* and *get3Δ* cells showed an increase in insoluble proteins compared to unstressed BY4741 and also heat-shocked BY4741 cells (Fig. 1D; Supplementary Fig. S2A, S2B online). Deletion of genes corresponding to the three other described components of the GET pathway, *GET4* (*YOR164C)*, *GET5* (*YOL111C*) and *SGT2* (*YOR007C*), also caused an accumulation of Hsp104-GFP foci (Supplementary Fig. S1B, S1C online). Complementation of the *get3Δ* phenotype using the corresponding MOBY plasmid confirmed that the phenotype is caused by deletion of *get3Δ* (Supplementary Fig. S2C, S2D online). Moreover, the protein levels of Hsp104-GFP were similar between BY4741 and *get3Δ* cells and slightly elevated in *get2Δ* cells (Supplementary Fig. S2E,F online), demonstrating the aggregate formation was not a result of diminished Hsp104 levels.

The GET pathway is responsible for insertion of proteins with a C-terminal transmembrane domain, i.e. tail-anchored proteins, into the ER membrane^20,21^ (Fig. 1E). As accumulation of Hsp104-GFP foci during non-stress conditions could be a phenotype shared by ER membrane insertion mutants, mutants of the SND pathway (*snd1Δ*)^22^ and the SRP-independent pathway (*sec72Δ*)^23^ were tested for accumulation of Hsp104-associated aggregates. However, the percentage of cells displaying Hsp104-GFP foci was similar in *snd1∆* mutants and wild-type (WT) cells (BY4741 Hsp104-GFP), while aggregate levels were slightly higher in *sec72Δ* cells (Supplementary Fig. S2G online).

Ubc9ts-GFP is known to misfold and aggregate at temperatures above 30 °C. When introduced into *get3Δ* cells, Ubc9ts-GFP formed foci even at 30 °C (Fig. 1F,G). This was further confirmed biochemically as Ubc9ts-GFP was enriched in the protein pellet fraction of *get2Δ* and *get3Δ* cells (Supplementary Fig. S2H,I online). Another misfolding-prone protein, gus1-3, formed foci at 30 °C in both *get2Δ* and *get3Δ* cells to a greater degree than in wild type cells^24,25^ (Fig. 1H,I). This shows that other proteins than TA proteins aggregate in GET mutants, indicating a general collapse in cytosolic proteostasis.

### Deletion of *get2* affects the folding of functionally and physically diverse proteins

**Figure 2:**
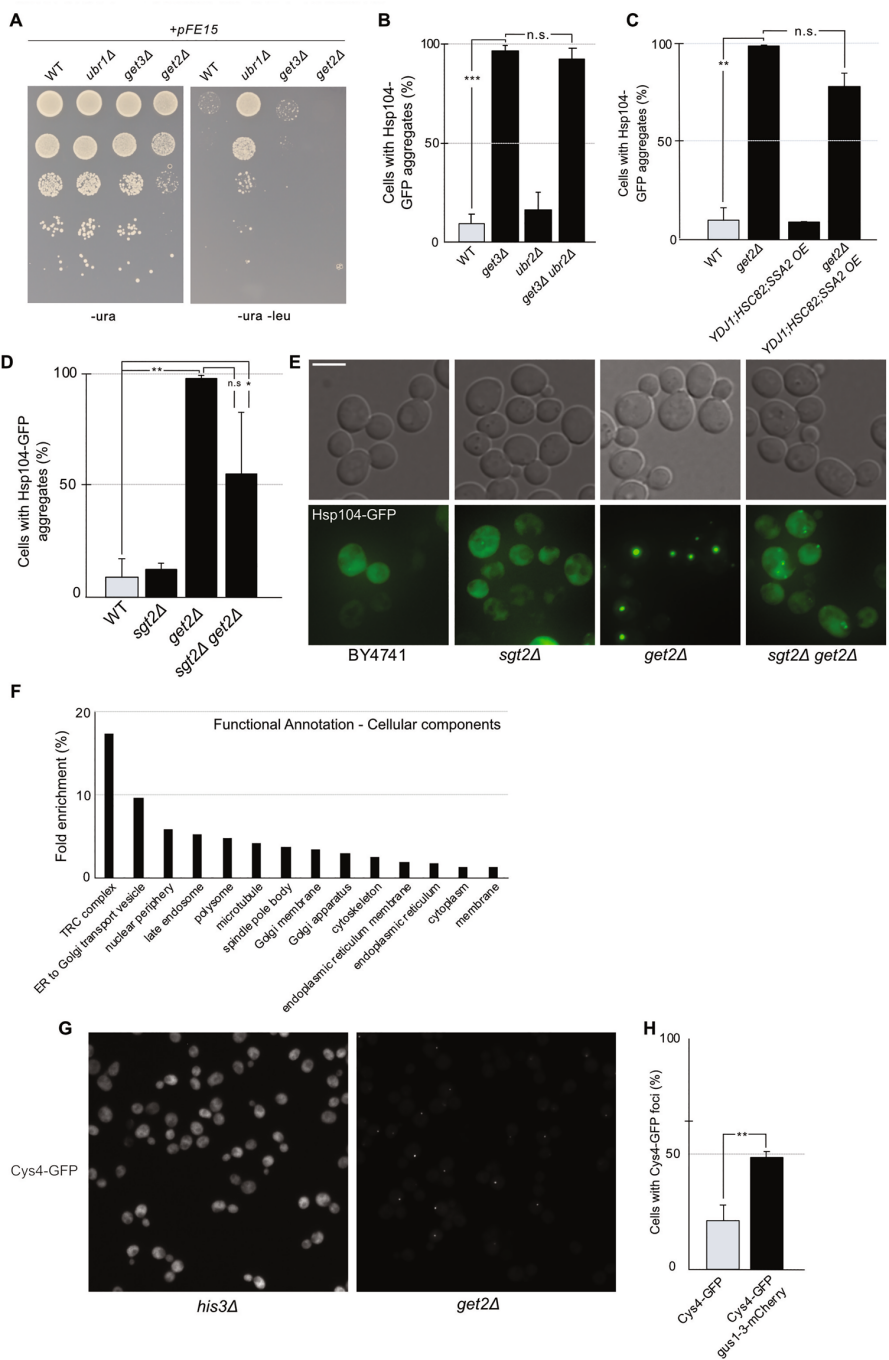
Mislocalized TA proteins are not solely responsible for the accumulation of aggregates in GET mutants. (**A**) Growth of BY4741, *ubr1Δ*, *get3Δ* and *get2Δ* cells carrying ΔssCPY*-Leu2. Strains expressing ΔssCPY*-Leu2 from a plasmid (pFE15) were spotted on CSM-ura and CSM-leu-ura plates in a four-fold dilution series and allowed to grow for 2 days at 30 °C (N = 3). (**B**) Hsp104-GFP aggregate load in wild type cells and mutant cells as indicated grown in midlog phase at 30 °C. Bar graphs show the percentage of mother cells with ≥ 1 Hsp104-GFP foci (N = 3, n > 200 cells per strain per replicate). (**C**) Percentage of mother cells with Hsp104-GFP aggregates in *get2Δ* cells overexpressing chaperones *YDJ1*, *SSA2*, and *HSC82* (N = 2, n > 200 cells per strain per replicate).(**D**) Hsp104-GFP aggregate load in *get2Δ sgt2Δ* cells in midlog phase at 30 °C. Bar graphs show the percentage of mother cells with ≥ 1 Hsp104-GFP foci (N = 3, n > 200 cells per strain per replicate). (**E**) Representative images of Hsp104-GFP foci in BY4741, *sgt2Δ*, *get2Δ*, *sgt2Δ get2Δ* cells displayed. GFP channel images displayed as maximal Z projection. (**F**) Functional enrichment of the hits from the GFP foci screen using Database for Annotation, Visualization and Integrated Discovery (DAVID, https://david.ncifcrf.gov/). Only significantly enriched (p > 0.05) GO annotations in Cellular Components are presented. (**G**) Images of *his3Δ* Cys4-GFP and *get2Δ* Cys4-GFP acquired using high content microscopy. (**H**) Cys4-GFP foci in cells carrying a misfolding reporter (*gus1-3-mCherry*). Bar graphs show the percentage of mother cells with ≥ 1 Cys4-GFP foci (N = 3, n > 200 cells per strain per replicate). Bar graphs are displayed as mean ± SD. * p < 0.05, ** p < 0.01, *** p < 0.001, n.s. > 0.05, unpaired two-tailed t test. Scale bar 5 µm.

Degradation of aberrant proteins via the UPS system is an essential branch of temporal protein quality control. Indeed, the transcriptional activator of proteasome genes, Rpn4 and the only non-essential 20S proteasome subunit, Pre9, were identified as hits in our screen (Supplemental table S1 online), suggesting that limited proteasome activity indeed promotes protein aggregation. Moreover, deletion of *PRE9* in *get3Δ* and *get2Δ* cells caused a growth defect in the double mutant cells (Supplemental Fig. S3A online). Therefore, one possible explanation for the accumulation of aggregated proteins in GET mutants could be a compromised UPS system. The efficiency of proteasomal degradation in mutants of the GET pathway was probed using a misfolded and cytosolic form of CPY (Prc1) linked to Leu2^26^. In brief, the protein quality control system will recognize the misfolded CPY-Leu2 and degrade it. Therefore, a yeast strain with efficient proteasomal degradation will be unable to grow in the absence of leucine (or grow poorly), while a strain with less efficient proteasomal degradation will be able to grow. However, proteasomal capacity appeared only limited in *get3Δ* cells and the data did not support a general link between aggregate accumulation and UPS activity in the GET mutants analyzed (Fig. 2A; Supplemental Fig. S3B online). Additionally, increasing the levels of proteasome subunits by deleting the cytoplasmic E3 ubiquitin ligase *ubr2*^27,28^ did not affect aggregate levels in *get3Δ* cells (Fig. 2B) and did not affect growth (Supplemental Fig. S3C online). In addition, simultaneous overexpression of *SSA2*, *YDJ1*, and *HSC82* in *get2Δ* cells had no significant effect on the Hsp104-GFP aggregate formation (Fig. 2C) or growth at 30 °C (Supplemental Fig. S3D online).

Another possible explanation for the presence of Hsp104-GFP foci in GET mutants might be TA protein mislocalization; disruption of the GET pathway has been shown to cause mislocalization of the TA protein Sed5p and its co-localization with Hsp104 and other chaperones^10,29^. On the other hand, it is possible that compromising GET activity causes aggregation of other non-TA proteins. Previous studies have shown that deletion of *SGT2* in *get2Δ* cells will remove GET-TA aggregates from the cytosol as the additional deletion of *SGT2* in the *get2Δ* background causes a decrease in TA loading onto Get3, which is dependent on Sgt2^30,31^. However, while the additional deletion of *SGT2* partially suppressed the aggregation phenotype of the *get2Δ* mutant, most Hsp104-associated aggregates still remained in the cytosol (Fig. 2D,E). This suggests that while TA proteins contribute to some protein aggregation, other proteins are also aggregating. Interestingly, deleting *sgt2* in *get2Δ* cells, did alleviate the growth defect seen in *get2Δ* cells (Supplemental Fig. S3E online).

To test which proteins were forming aggregates in GET-deficient cells, another genome-wide screen was performed by introducing deletion of *GET2* or *HIS3* (WT control) in the yeast GFP collection to create two matching libraries. The cells were grown to mid-exponential phase at 30 °C and the localization of the GFP signal was compared between the two libraries (Supplementary Fig. S3F online). The list of hits (proteins that formed foci in *get2∆* mutant but not wild type cells; Supplementary Fig. S4; Supplemental table S2 online) was analyzed for Gene Ontology (GO) enrichment using the online tool DAVID^32,33^. (Fig. 2F). GO enrichment for cellular components found an expected enrichment for the cellular component “TRC complex” (GO term GO:0072380; Get4, Get5, Sgt2) but several other cellular components/processes were also enriched among the hits; for example, “protein retention in ER lumen” and other processes unrelated to TA insertion and the GET pathway (Supplementary Fig. S3G online). Additionally, the percentage of hits with predicted transmembrane domains (21%; Supplementary Fig. S3H online) closely matched previous reports for the entire *Saccharomyces cerevisiae* proteome^34^, indicating no bias for transmembrane proteins among the foci-forming proteins. Moreover, no bias was found for molecular weight (Supplementary Fig. S3I online). Other protein characteristics that could possibly have an impact on aggregation propensity, for example the presence of prion-like domains (PrLDs) and intrinsical disorderedness, were also analyzed among the hits (Supplemental table 2 online). While PrLDs are often found in aggregation-prone proteins, only three proteins among the hits were predicted to contain a PrLD using the online tool PrionW (http://bioinf.uab.cat/prionw/)^35,36^. However, 24% of the hits were found to be intrinsically disordered using MobiDB (https://mobidb.bio.unipd.it/; a protein is considered significantly disordered if the glutamine/asparagine content is more than 20% with a pWalz cutoff of 73,55)^37^. Previous studies have predicted 7.7% intrinsically disordered proteins in the yeast proteome^38^, indicating that intrinsically disordered proteins were enriched in our screen.

Some hits were expected to form foci as part of a known stress response (e.g. Hsp104p, Ssa1p, Btn2p, Sis1p; Supplementary Fig. S4A–D; Supplemental table S2 online). The presence of foci of Btn2-GFP could be explained by the increased Hsf1 activity found in *get2Δ* mutant cells (Supplementary Fig. S3J online). Some hits were associated with previously reported GET pathway-related foci (Get1p, Get5p and Sgt2p; Supplementary Fig. S4E; Supplemental table S2 online)^29^ whereas others had functions unrelated to stress responses, e.g. Cys4p, which functions as a cystathionine beta-synthase^39^ (Fig. 2G; Supplemental table S2 online). Cys4-GFP was previously reported among metabolic proteins that form condensates during the post-diauxic shift and in stationary phase^40^. Cys4-GFP was manually confirmed to form foci in both *get2Δ* and *get3Δ* cells (Supplementary Fig. S3K online). Moreover, GFP tagged Cys4 activity was functionally active as it was growing similar to wild type cells at 37 °C heat stress while *cys4Δ* cells were severely affected (Supplementary Fig. S3L online). Metabolic proteins forming condensates were also shown to recruit Hsp104p and Ssa1p, indicating a role for the protein quality control system in regulating the solubility of metabolic condensate proteins^40^. Indeed, introducing folding stress and added pressure on the proteostasis system by introducing the misfolding reporter gus1-3-mCherry^24,25^, in a wild type strain carrying Cys4-GFP, resulted in Cys4-GFP forming foci to a greater degree than in a wild type strain carrying only Cys4-GFP (Fig. 2H). This suggests that the Cys4-GFP foci identified in *get2Δ* cells could be a consequence of a heavy burden on the protein quality control system limiting the capacity to maintain overall protein solubility in the cell.

### GET pathway deficiency alters spatial protein quality control

**Figure 3:**
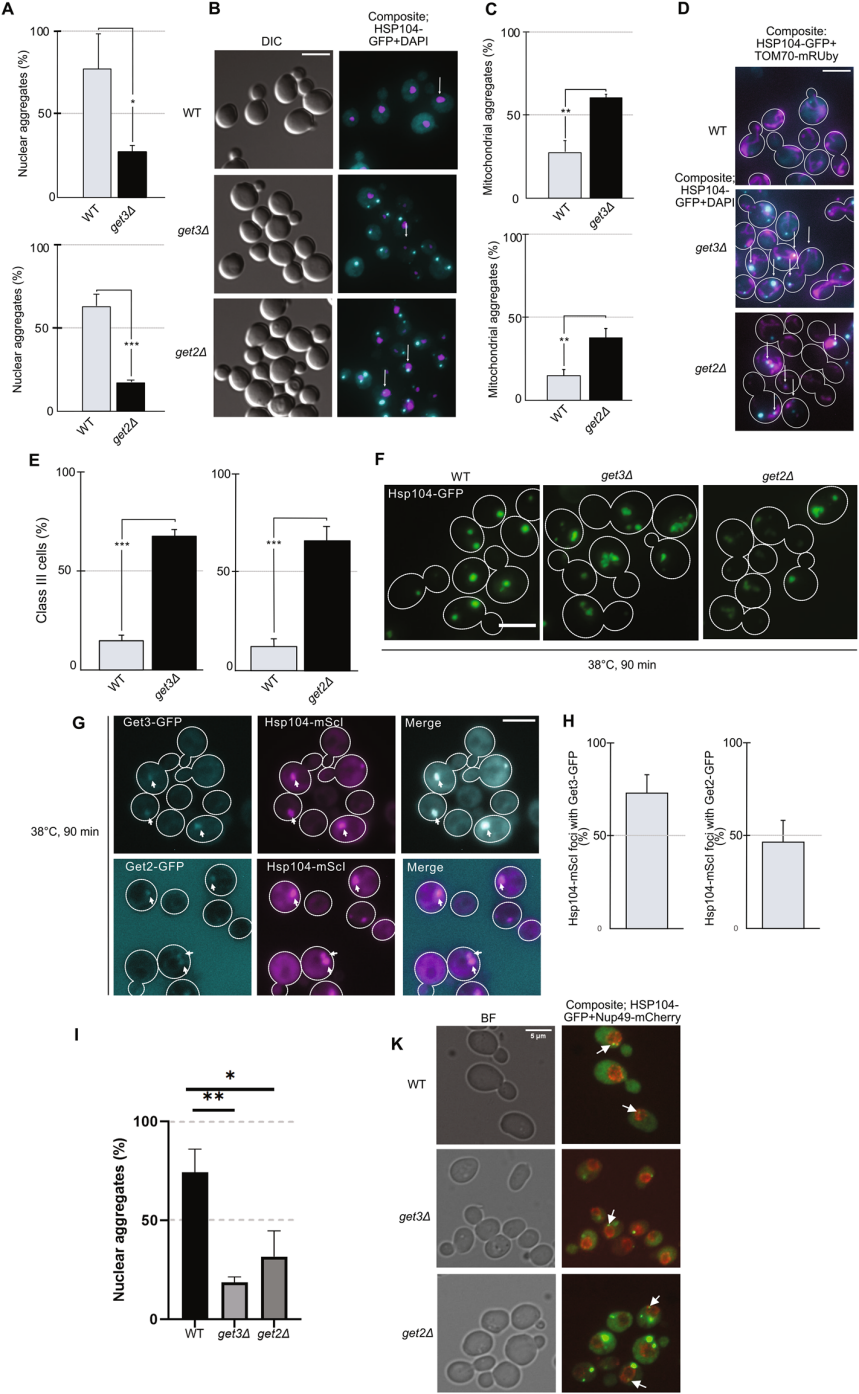
GET mutants display defects in spatial protein quality control and are sensitive to protein misfolding stress. (**A**) Localization of Hsp104-GFP aggregates with respect to the nucleus in *get3Δ* and *get2Δ* cells in midlog phase at 30 °C. Bar graphs show the percentage of aggregates in the nucleus using Hsp104-GFP and DAPI (N = 3, n > 200 cells per strain per replicate). (**B**) Representative DIC and composite fluorescent images displaying Hsp104-GFP (cyan) and DAPI (magenta). GFP and DAPI channels displayed as a composite of maximal Z projections of both channels. Arrows show aggregates near the nucleus. (**C**) Localization of Hsp104-GFP aggregates with respect to mitochondria in *get3Δ* and *get2Δ* cells in midlog phase at 30 °C. Bar graphs show the percentage of aggregates in mitochondria using Hsp104-GFP and TOM70-mRuby as reporters (N = 3, n > 194 per strain per replicate). (**D**) Representative composite fluorescent images displaying Hsp104-GFP (cyan) and TOM70-mRuby (magenta). GFP and mRuby channels displayed as a composite of maximal Z projections of both channels. Arrows show aggregates at mitochondria. (**E**) Inclusion formation in *get3Δ* and *get2Δ* cells after heat shock at 38 °C for 90 min. Bar graphs show the percentage of class III cells (cells with > 2 Hsp104-GFP foci; N = 3, n > 200 cells per strain per replicate). (**F**) Representative images of inclusion formation of Hsp104-GFP in BY4741, *get3Δ* and *get2Δ* cells. GFP channel images displayed as maximal Z projection. White outlines show mother cells and daughter cells. (**G**) Representative images of Hsp104-mScarletI (displayed as magenta) foci colocalizing with Get3-GFP (cyan; upper panel) and Get2-GFP (cyan; lower panel). (**H**) Quantification of percentage of Hsp104-mScarletI foci colocalizing with Get3-GFP (left graph) and Get2-GFP (right graph) (N = 3, n = 200 cells analyzed for each strain). (I) Similar to Fig. 3A, Hsp104-GFP aggregates localizing near the nucleus were monitored with mCherrey-tagged Nup49. Bar graph shows percentage of Hsp104-GFP aggregates at the nucleus (N = 3, n = 200 cells analyzed for each strain). (K) Representative Bright Field and composite fluorescent images displaying Hsp104-GFP (green) and Nup49-mCherry (Bright Red). Bar graphs are displayed as mean ± SD. * p < 0.05, ** p < 0.01, *** p < 0.001, n.s. > 0.05, unpaired two-tailed t test. Scale bar 5 µm.

The term spatial quality control is used to describe how cells handle misfolded proteins by sequestering such proteins in spatially distinct aggregates and inclusions in the cell. One such inclusion is the intranuclear/perinuclear INQ/JUNQ site^41,42^. When WT cells grow in mid-exponential phase at 30 °C, the majority of the few Hsp104-GFP foci present (found in less than 4% of the cells; Supplementary Fig. S5A,B online) were localized near the DAPI stained nucleus (Fig. 3A,B). These results were further confirmed where a nuclear envelope protein was tagged with mCherry (Fig. 3I,K). In contrast, Hsp104-GFP foci in *get3Δ* and *get2Δ* cells were distant to the nucleus (Fig. 3A,B) and generally close to mitochondria (Fig. 3C,D).

Normally WT cells will form 1–2 inclusions upon a 90 min heat shock at 38 °C^8,10,41^. Failure to do so, i.e. when cells have 3 or more inclusions, is referred to as an inclusion formation defect or class III phenotype^10^. For *get2Δ* and *get3Δ* cells, an inclusion formation defect was found in significantly more cells than for WT cells after 90 min at 38 °C (Fig. 3E,F). This defect was reversed by reintroducing a copy of *GET3* in *get3Δ* cells, confirming that this defect was specifically caused by the *GET3* deletion (Supplementary Fig. S5C,D online). An inclusion formation defect was also found in *get1Δ*, *get4Δ*, *get5Δ* and *sgt2Δ* cells (Supplementary Fig. S5E,F online).

Previous studies have described Get3p as a holdase chaperone that forms foci during glucose starvation. These foci contained Sed5p and colocalized with chaperones Sis1p, Ssa2p and Hsp42p^29^. To further study a role for Get3p in protein quality control during heat stress, cells expressing both Get3-GFP and Hsp104-mScarletI were exposed to a 90 min continuous heat shock at 38 °C. Before heat shock both Get3-GFP and Hsp104-mScarletI signals were largely diffuse and cytosolic (Fig. 3G). The continuous heat shock induced the expected Hsp104-mScarletI foci formation^43^ (Supplementary Fig. S5G online) and around 70% of these foci also contained Get3-GFP (Fig. 3G,H). Similarly, around 46% of Hsp104-mScarletI foci formed after heat shock colocalized with Get2-GFP (Fig. 3G,H). This shows that other GET components, other than Get3, can be of importance for proteostasis during heat shock.

### GET pathway mutants are sensitive to protein misfolding stress and misfolding disease proteins

**Figure 4:**
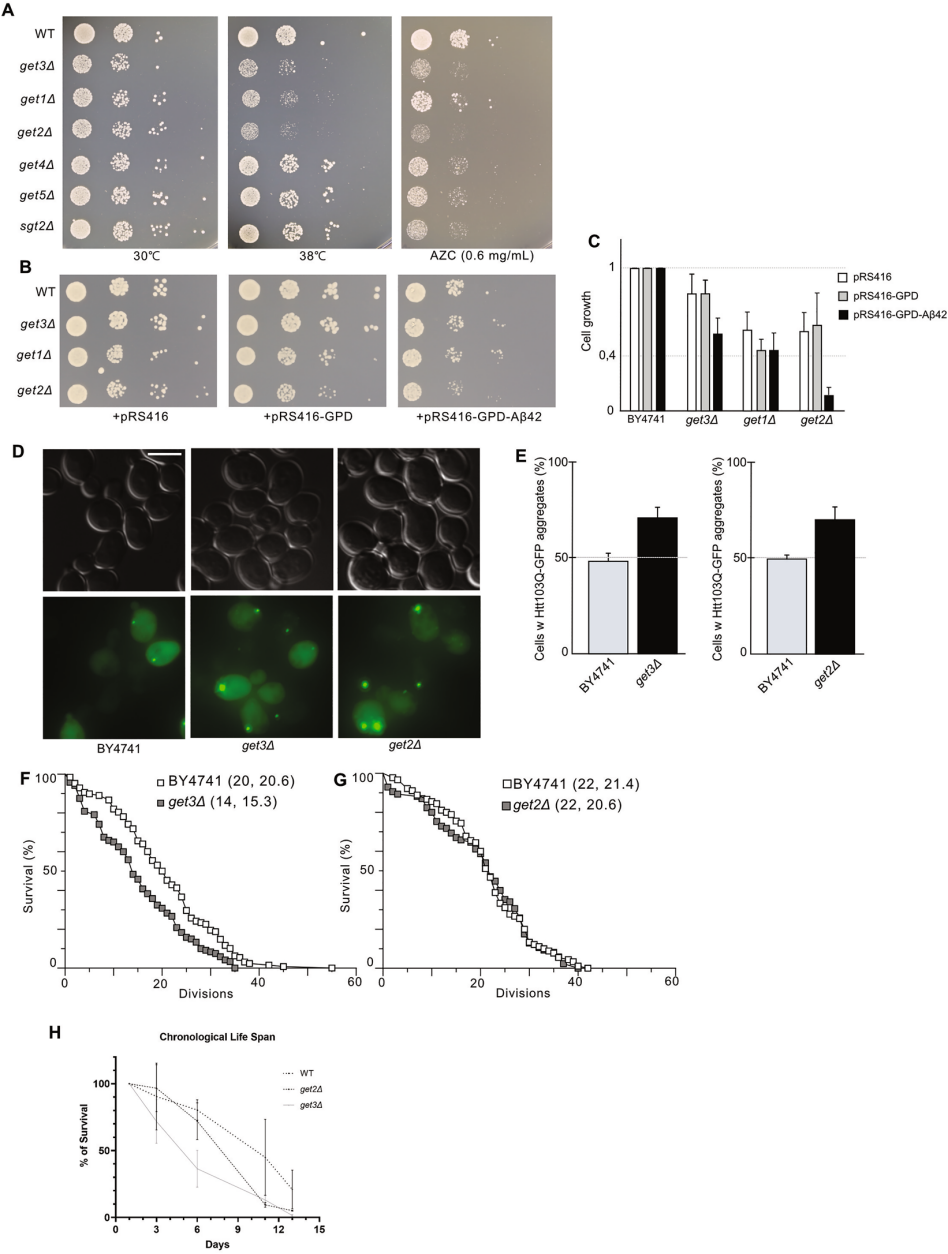
(**A**) Growth of GET mutants at 30 °C, and 38 °C, and in the presence of 0.6 mg/mL AZC (at 30 °C). Cells were spotted onto YPD or YPD + 0.6 mg/mL AZC plates in four-fold dilution series and incubated for 2 days before being imaged (N = 3). (**B**) Growth test of *get3Δ*, *get1Δ*, and *get2Δ* carrying plasmids expressing Aβ42. Cells were spotted onto CSM-ura plates in four-fold dilution series and incubated for 2 days at 30 °C before being imaged (N = 3). (**C**) Quantification of the Aβ42 growth test. Growth was measured and normalized to BY4741 carrying the corresponding plasmid (N = 3). (**D**) Representative images of mHtt103Q-GFP in BY4741, *get3Δ* and *get2Δ* cells. GFP channel images displayed as maximal projections. (**E**) mHtt103Q-GFP aggregate load in BY4741, *get3Δ* and *get2Δ* cells. Bar graphs show the average percentage of cells with mHtt103Q-GFP foci (N = 3, n > 200 cells per strain per replicate). Bar graphs are displayed as mean ± SD. (**F**, **G**) Replicative lifespan determined for *get3Δ* (n = 120, wild type n = 128; p < 0.0001) and *get2Δ* (n = 85, wild type n = 90; n.s.). * p < 0.05, ** p < 0.01, *** p < 0.001, n.s. > 0.05, unpaired two-tailed t test. Scale bar 5 µm. (**H**) Chronological lifespan of BY4741, *get2Δ*, *get3Δ* cells. The curves show the average percentage of survival after day 1 of culture from three replicates (N = 3).

The three hits from the unstressed screen (*get2Δ*, *get3Δ* and *get1Δ* cells) were also all found to grow poorly at 38 °C (Fig. 4A), a temperature that causes protein misfolding. Additionally, *get2Δ* and *get3Δ* cells were also sensitive to the proline analog AZC, which causes misfolding as it is incorporated into polypeptides during translation (Fig. 4A). In contrast, *get1Δ* cells as well as the mutants of the upstream GET components (*sgt2Δ*, *get4Δ and get5Δ)* were less sensitive to AZC than *get1∆-3∆* cells (Fig. 4A). The reason for this differential sensitivity of different *get* mutants to translation errors is not clear.

Alzheimer’s disease and Huntington’s disease are two protein conformational disorders in humans, directly connected to the accumulation of aggregated peptides. The amyloid-β peptide (Aβ) is generated in human cells when the amyloid precursor protein (APP) is sequentially cleaved. The disease-associated Aβ42 peptide, is more aggregation-prone than the shorter Aβ40 peptide, which is associated with healthy neurons. In yeast, Aβ42 can be constitutively expressed from a plasmid and this peptide causes modest toxicity^44^. We found that this disease protein caused greater toxicity in *get2Δ* cells than in control cells (Fig. 4B,C), in agreement with previously reported results^44^. Similarly, the polyQ-expanded form of Huntingtin exon 1, Htt103Q, can be expressed in yeast and will form amorphous aggregates, causing toxicity in WT cells^45,46^. Expression of Htt103Q caused an increased Htt103Q aggregate load and aggregate size in *get3Δ* and *get2Δ* cells (Fig. 4D,E).

Accumulation of misfolded and aggregated proteins is considered a hallmark of aging^3,4,47^. Just as *get3Δ* cells carry Hsp104-GFP aggregates during the exponential growth phase, the aggregate load was higher in replicatively old *get3Δ* cells (median age 12–14 generations) than in wild type cells (Supplementary Fig. S6A online). Given the established role for protein aggregation in aging, we set out to determine if the increased aggregate burden and limited protein folding capacity of *get2Δ* and *get3Δ* cells was associated with changes in the rate of aging, both replicative^48^ and chronological aging^49^ (survival of growth-arrested, stationary phase cells). The *get3Δ* cells displayed a shorter median replicative lifespan compared to WT cells (median age 14 generations for *get3Δ* cells, median age 20 generations for WT control cells; Fig. 4F) whereas the lifespan of *get2Δ* cells was similar to that of wild type cells (median age 22 generations for both strains; Fig. 4G). This suggests that there is no obvious link aggregate load and replicative lifespan in these two *get* mutants. However, both *get* mutants displayed a shorter chronological life span than WT cells which could be related to the increased aggregate burden of chronologically aged *get* mutant cells (Fig. 4H).

## Discussion

A screen examining bottlenecks in protein folding might be expected to yield hits relating to temporal protein quality control, as this system encompasses both protein folding and protein degradation. However, apart from *RPN4*, the transcriptional regulator of the proteasomal genes^27^, and *PRE9*, the only non-essential 20S proteasome subunit^48^, no other hits related to temporal protein quality control were identified in the screen. This could be explained by the high degree of redundancy in the system, which is both direct, e.g. yeast has four homologs of the major cytosolic Hsp70, *SSA1-4*, and indirect in that deletion of a proteostasis component will cause compensatory upregulation of other genes. Indeed, disrupting Hsp70 redundancy by deleting both *SSA1* and *SSA2* or introducing a temperature sensitive allele of *SSA1* as the only cytosolic Hsp70 present (*ssa1ts ssa2Δ ssa3Δ ssa4Δ*) does cause Ubc9ts-GFP aggregation even at permissive temperatures^50^. Additionally, deletion of both *SSA1* and *SSA2* will cause compensatory expression of the otherwise heat stress-inducible *SSA4*^51^. Another important aspect is that our screen only covered the yeast knockout library and no essential genes were considered, e.g. proteasomal subunits. Extending the same screening concept to include the temperature sensitive alleles of essential genes would indicate more cellular processes that constitute bottlenecks for protein folding.

Others have shown that disruption of the GET pathway will cause accumulation of aggregated GET pathway components and TA proteins in the cytosol^29^. These aggregates also colocalized with proteostasis components such as Hsp104. Our screen searching for proteins that aggregated in *get2Δ* cells demonstrated that proteins unrelated to protein quality control and TA proteins formed foci as a result of GET deficiency: e.g. the cystathionine beta-synthase Cys4-GFP. It is known that several proteins with a role in metabolism are capable of forming foci or condensates upon metabolic shifts, such as post-diauxic shift and starvation^40^. The effect seen on non-TA proteins could be an indirect effect caused by aggregation of TA proteins and lack of TA protein function. It can also, however, be a direct effect of limited Get3 availability. Get3 has been described as a holdase chaperone during oxidative stress conditions, suggesting that a role for Get3 in other conditions is possible and could possibly affect non-TA protein solubility^52^. Interestingly several of the foci-forming proteins in *get2Δ* cells were also identified in a screen for condensate formation upon metabolic shifts of 440 proteins that previously have been annotated in metabolic processes^40^ (Supplemental table 2 online). 24% of the proteins was found to contain intrinsically disordered regions. Previous studies have found that around 7.7% of the *Saccharomyces cerevisiae* proteome has some degree of disorder^38^. Thus there is a bias for intrinsically disordered proteins among foci-forming proteins in the *get* mutant analyzed. This indicates that intrinsic disorder is of relevance for protein folding and solubility when proteostatic capacity is limited, just as it is of relevance in proteins that phase-separate in vitro^53^. It has been suggested that protein quality control components are involved in regulating formation of phase-separated condensates, such as those formed by Cys4^40^. Additionally, we found that challenging chaperone availability and proteostasis capacity by introducting a misfolding reporter, gus1-3-mCherry, affects the solubility of Cys4-GFP and causes its aggregation. This suggests that protein folding capacity may be limited in *get2Δ* cells, causing Cys4-GFP to form foci, which is in line also with the increased aggregation tendency of both gus1-3-GFP and Ubc9ts-GFP and the increased sensitivity of *get3Δ* and *get2Δ* cells to Aβ42 and Htt103Q.

As a complimentary cellular strategy to temporal protein quality control, spatial protein quality control acts to sort misfolded and aggregated proteins to specific sites in the cell. While a nuclear site (INQ, JUNQ) and a vacuolar site (IPOD) have been defined^41^, mitochondria have also been suggested to take part in handling misfolded proteins in both yeast and HeLa cells^54,55^. Sequestration of protein aggregates in *get3Δ* and *get2Δ* cells were associated with mitochondria rather than the nucleus, which is the dominant inclusion site in unstressed wild type cells. The fact that the misfolded proteins are found at mitochondria rather than in the nucleus could be a reflection of TA proteins becoming sorted to the mitochondria rather than the ER when the GET pathway is compromised, as has been previously described^21,56^. Moreover, Get3-GFP and Get2-GFP were both found to co-localize with Hsp104-mScarletI after heat shock. This could be because the two proteins have a functional role in protein quality control, which has already been suggested for Get3^29^. Another possibility is that Get3 and/or Get2 are inherently localized to sites of extensive proteins misfolding as studies have found that the ER surface is a major site for protein misfolding upon heat shock^57^.

We found that cells defective in the GET pathway display differential sensitivity to protein misfolding stress, e.g. elevated temperatures and exposure to AZC. It has been highlighted previously that the diverse phenotypes caused by deleting different *GET* genes can point to the fact that each component may have different roles beyond membrane insertion of TA proteins^21^. Indeed, we found that *get3Δ* cells were less efficient in degrading a proteasomal substrate (cytosolic misfolding Prc1p) than other *get* mutants, indicating differential roles of Get components on the UPS. It should be noted also that mutants of the Get pathway have previously been reported to readily accumulate suppressor mutations^21^, which might explain the differential phenotypes of *get* mutants. Moreover, studies have shown that the absence of one of the ER membrane receptor subunits will cause instability in the other. For example *get1* deletion results in lower Get2 protein levels^21^. Similarly, expression of the mammalian counterparts, WRB and CAML, in a yeast system, showed stabilization of the respective binding interaction partner upon co-expression of the other^58^.

We report here that the GET pathway appears to be a bottleneck in protein quality control in yeast cells, as single mutations in this pathway result in extensive protein aggregation. The data indicate that the pathways is poorly (genetically) buffered but to what extent a decline in the functionality of this pathway is a cause of protein aggregation during cellular aging remains to be analyzed. However, we found that some specific deletions in genes of the GET pathways affects the rate of both replicative and chronological aging. Analyzing whether the conserved GET pathway is a bottleneck also in the protein quality control of higher eukaryotes and subjected to a functional decline during aging may be warranted.

## Methods

### Strains, plasmids, and growth conditions

The *Saccharomyces cerevisiae* yeast strains are described in detail in Supplementary table 3 online. Cells were grown in yeast extract peptone dextrose (YPD; 2% peptone, 1% yeast extract, 2% glucose) or dropout medium (for plasmid strains) lacking the corresponding plasmid selective marker, unless otherwise stated. Strains were grown at 30 °C and liquid cultures were stirred at 180 rpm, unless otherwise stated. Strains were constructed using LiAc-treated yeast cells transformed with linear DNA or plasmid DNA that were plated on plates with appropriate selection agents. All transformants were confirmed using PCR (primer details are listed in Supplementary table 4 online).

### Large-scale high content microscopy-based screening

The large-scale screens were done in the S228C SGA background, while manual confirmation of screen hits was done in BY4741 background. All other experiments were performed in BY4741 background with deletion mutants from the YKO collection (EUROSCARF). A library was constructed using SGA technology^59–61^ to introduce *HSP104-GFP-LEU2* into the yeast deletion library (Y7092 background)^8^. Cells were grown in SC-leu medium supplemented with G418 antibiotic to midlog phase and then fixed with 3.7% formaldehyde. The fixed cells were imaged using the high content microscope ImageXpress Micro XLS system (Molecular devices, San Jose, California, USA) equipped with a 100X objective (CFI L Plan EPI cc 0 mm to 0.7 mm) and GFP filter (excitation 472/30 nm, emission 520/35 nm, dichroic mirror 495 nm) and scored for the percentage of mother cells with Hsp104-GFP foci.

Another library was constructed by crossing the *get2Δ::kanmx* (Y7093 background) query strain into the yeast GFP library (*XXX-GFP::his3mx6*). A control library was also constructed by crossing the *his3Δ::kanmx* cassette into the yeast GFP library. Cells were grown to midlog phase in YPD medium (2% glucose) and then fixed by formaldehyde treatment. GFP signal was recorded using high content microscopy ImageXpress Micro XLS and the signal pattern was manually compared between the *get2Δ::kanmx XXX-GFP* library and the control library. A comparison was considered a hit if the GFP signal pattern was different (Supplementary Fig. 3F online) in a given GFP strain between the two libraries. The GFP strain was considered a hit if the signal was cytoplasmic in the control and in foci in *get2Δ* cells, in more or larger foci in *get2Δ* cells than in control cells or if the signal was in a structure in the control library and in a similar structure as well as in foci in the *get2Δ* library. Strains with an obviously mitochondrial signal were excluded from the analysis as *get2Δ* cells are reported to have abnormal mitochondrial morphology with partially fragmented mitochondria that can be mistaken for foci formation^56^. In addition, if a given strain failed to grow in either or both libraries, that GFP strain was also excluded from the analysis. Hits from both screens were processed using the STRING database (https://string-db.org/), SAFE analysis (thecellmap.org), and functionally enriched using Database for Annotation, Visualization and Integrated Discovery (DAVID, https://david.ncifcrf.gov/)^32,33^. Hits were analyzed for predicted transmembrane helices using TMHMM—2.0 (https://services.healthtech.dtu.dk/service.php?TMHMM-2.0)^34,62^.

### Fluorescence microscopy

Images were acquired using a Zeiss Axio Observer.Z1 inverted microscope with Apotome and Axiocam 506 camera (Carl Zeiss AB) with a Plan-Apochromat 100x/1.40 Oil DIC M27 objective (Carl Zeiss AB). The microscope is equipped with the following filters: 38 HE Green Fluorescent Protein λEx.: BP 470/40, Beamsplitter: FT 495, λEm.: BP 525/50. 45 Texas Red, λEx.: BP 560/40, Beamsplitter: FT 585, λEm.: BP 630/75.49 DAPI, λEx.: G 365, Beamsplitter: FT 595, λEm.: BP 445/50. For aggregate load experiments cells were grown to midlog phase in YPD at 30 °C. A sample of live cells was centrifuged and imaged immediately. For heat shock experiments, the remaining cultures were heat shocked at 38 °C for 90 min, with live cells being imaged immediately. Aggregate load was scored as the percentage of mother cells with foci of a given aggregate or misfolding reporter (Hsp104-GFP, gus1-3-GFP, Cys4-GFP or Ubc9ts-GFP). After heat shock, inclusion formation deficiency was scored as the percentage of cells forming more than two Hsp104-GFP foci. For Ubc9ts-GFP experiments cells with pUbc9ts-GFP^41^ were grown to early log phase (OD_600_ 0.2) in SC-ura + 2% raffinose at 30 °C. Expression was induced by incubation with 2% galactose for 5 h at 30 °C and live cells then imaged using fluorescence microscopy. For experiments with DAPI staining, cells were grown to midlog phase in YPD at 30 °C and fixed with formaldehyde treatment (3.7% formaldehyde for 30 min at room temperature). The cells were resuspended in PBS and incubated with DAPI for 10 min in the dark. Cells were then imaged.

### Western blot analysis

BY4741, *get2Δ* and *get3Δ* cells were grown overnight and diluted to OD_600_ 0.1 and grown to mid-exponential phase (OD_600_ 0.5) and proteins were extracted as described^14^. Extracted proteins were immunoblotted with anti-GFP (ab6556; Abcam, Cambridge, UK) and anti-Pgk1 (catalogue number 459250, clone 22C5D8; Invitrogen, Waltham, Massachusettes, USA) antibody. Hsp104-GFP levels were normalized to loading control (Pgk1) and compared to BY4741 (set to 1).

### Growth tests

Cells were grown to midlog (OD_600_ 0.4–0.5) in YPD or dropout medium (for plasmid strains) and then serially diluted tenfold for OD_600_ ranging from OD_600_ 10^−1^ to 10^−4^. The dilutions were then spotted onto agar plates and incubated 2–3 days at 30 °C (unless otherwise stated). For sensitivity to L-Azetidine-2-carboxylic acid (AZC)^63–66^ concentration of 0.6 mg/mL was used. For heat sensitivity, the plates were incubated at 38 °C. Growth tests of cells expressing ΔssCL*^26^ were done on SC-ura and SC-leu-ura plates. For growth tests in presence of human disease proteins the plasmid pRS416-GPD-Aβ42 was a gift from Dina Petranovic^44^ and the pRS416 Htt103Q GPD plasmid was a gift from Susan Lindquist via Addgene (Addgene plasmid number 1180, https://www.addgene.org/1180/, RRID:Addgene_1180^46^.

### Cell fractionation assay

Cell lysate fractionation is performed as described^67^ with some modifications. Briefly exponentially growing yeast cells were lysed using silica beads using bead beater (Fastprep) 5.5 ms/s for 30 s for 4 cycles at 4 °C in 50 mM Tris pH 7.5, 150 mM NaCl, 5% Glycerol, 1X Protease Inhibitor (Pierce™ Protease Inhibitor Mini Tablets, catalogue number A32955; Thermo Fisher Scientific, Waltham, Massachusttes, USA) and Pefabloc. Cells were kept on ice for 1 min in between the cycles. Cell debris was removed by centrifuging the cells for 4000 rpm for 1 min. Protein concentration was measured using Bradford assay (Quick Start™ Bradford 1× Dye Reagent, catalogue number 5000205; Bio-Rad laboratories, Hercules, California, USA) and protein amounts were normalized before cell fractionation. Cell lysates were fractionated into supernatant (soluble) and pellet (insoluble) fractions by centrifuging cells at 13,000 rpm for 30 min at 4 °C. Pellet (insoluble) fractions were dissolved in reduced protein loading buffer (4× Laemmli Sample Buffer, catalogue number 1610747, Bio-Rad laboratories, Hercules, California, USA) and loaded onto Criterion XT Precast Gel 4–12% (Bio-Rad, Hercules, California, USA). Proteins were visualized with silver staining using Pierce Silver stain kit (Pierce™ Silver Stain Kit, catalogue number A32955, Thermo Fischer Scientific Waltham, Massachusettes, USA).

### HSF1 activity

The pAM09 and pAM10 plasmids^68,69^ were introduced into mutants of interest. Cells were then cultured in SC-ura medium with 2% glucose until midlog phase. NanoLuc detection was then performed as previously described using NanoGlo substrate (NanoGlo Luciferase Assay system, Promega GmBH, Germany)^69^ diluted 1:100 in the supplied substrate buffer and added in 1:10 dilution to the cells. The cells were then incubated 3 min and Bioluminescence was determined at 25 °C using a Synergy 2 microplate reader (BioTek, Winooski, VT, USA).

### Replicative lifespan analysis

Yeast replicative lifespan was analyzed using standard procedures^4,70^. In brief, exponentially growing cells (OD_600_ 0.3) were plated onto YPD agar plates and placed in a grid using a micromanipulator (Singer Systems, Watchet, UK). After one division, virgin mother cells were kept in the grid and daughter cells were removed and counted upon division.

### Chronological lifespan assay

Chronological Life Span is performed as described^71^ with some modifications. Yeast strains were grown in synthetic complete dextrose (SDC) medium overnight. 0.1 OD_600_ of cells were inoculated and grown in 20 mL in 100 SDC medium. On day 1, 10 μL cell suspension from flasks were diluted 10,000 times (BY4741, *get3Δ* and *get2Δ)*. 50 μl of the diluted cells were plated onto YPD plates. Plates were incubated at 30 °C for 2–3 days and colony forming units (CFU) were counted. This was repeated on days 3, 6, 11 and 13. The number of CFU on each day was compared to the number of CFU on day 1 (set to 100%).

### Old cell isolation—microscopy experiments

Aged cells were isolated using modified versions of previously established protocols^72,73^. In short, 50 mL cell cultures were grown to midlog phase at 30 °C and then labeled with 0.5 mg/mL EZ link sulfo-NHS-LC biotin (Thermo Fisher Scientific, Rockford, IL, USA). After excess biotin had been washed away, cells were resuspended in 200 mL YPD for overnight growth. The next day, cells were washed in PBS and incubated with 0.75 mg/mL MagnaBind Streptavidin beads (Thermo Fisher Scientific, Rockford, IL, USA) for 2 h at 4 °C. Aged cells were isolated using a magnetic sorter and washed in PBS + 0.5% glucose. The isolated cells were again resuspended in YPD for overnight growth and the procedure was repeated the next day. After the second isolation, part of the cells was used for imaging of Hsp104-GFP and the other part was stained with WGA (WGA-Alexa fluor 350, 10 μg/mL working concentration, Thermo Fischer Scientific) and imaged for determination of median age (i.e. number of bud scars).

### Statistical analysis

For microscopy experiments Microsoft Excel (version 16.46) was used to generate bar charts representing the mean values of three replicates of at least 200 cells, with error bars representing standard deviation. Two-tailed unpaired t tests were used to determine statistically significant differences between strains. For replicative lifespan analysis survival curves are shown, based on two replicates. Statistical testing was done using GraphPad Prism 7.01 and statistical significance was determined using unpaired Mann–Whitney U test. Any p values below 0.05 were considered to be significant.

## Supplementary Information

Supplementary Information.

## Data Availability

The image data sets generated in the high-content microscopy screens are not deposited in a public repository due to the large number of images acquired in each of the two screens. All data is available upon request and will be fulfilled by corresponding author Thomas Nyström (thomas.nystrom@cmb.gu.se).
