# Supplementary material for: The GET pathway is a major bottleneck for maintaining proteostasis in Saccharomyces cerevisiae
Source: Sci Rep. 2023 Jun 7;13:9285. doi: 10.1038/s41598-023-35666-8 (PMC10247811; doi:10.1038/s41598-023-35666-8)
Supplement: Supplementary file 1 — Supplementary Information. [file 41598_2023_35666_MOESM1_ESM.pdf]

SUPPLEMENTARY INFORMATION

The GET pathway is a major bottleneck for maintaining proteostasis in *Saccharomyces cerevisiae*

Rebecca Josefson[1], Navinder Kumar[1], Xinxin Hao[1], Beidong Liu[2], Thomas Nyström[1*]

[1] Department of Microbiology and Immunology, Institute of Biomedicine, Sahlgrenska Academy, University of Gothenburg, Gothenburg, Sweden

[2] Department of Chemistry and Molecular Biology, Faculty of Science, University of Gothenburg, Gothenburg, Sweden

*For correspondence: thomas.nystrom@cmb.gu.se

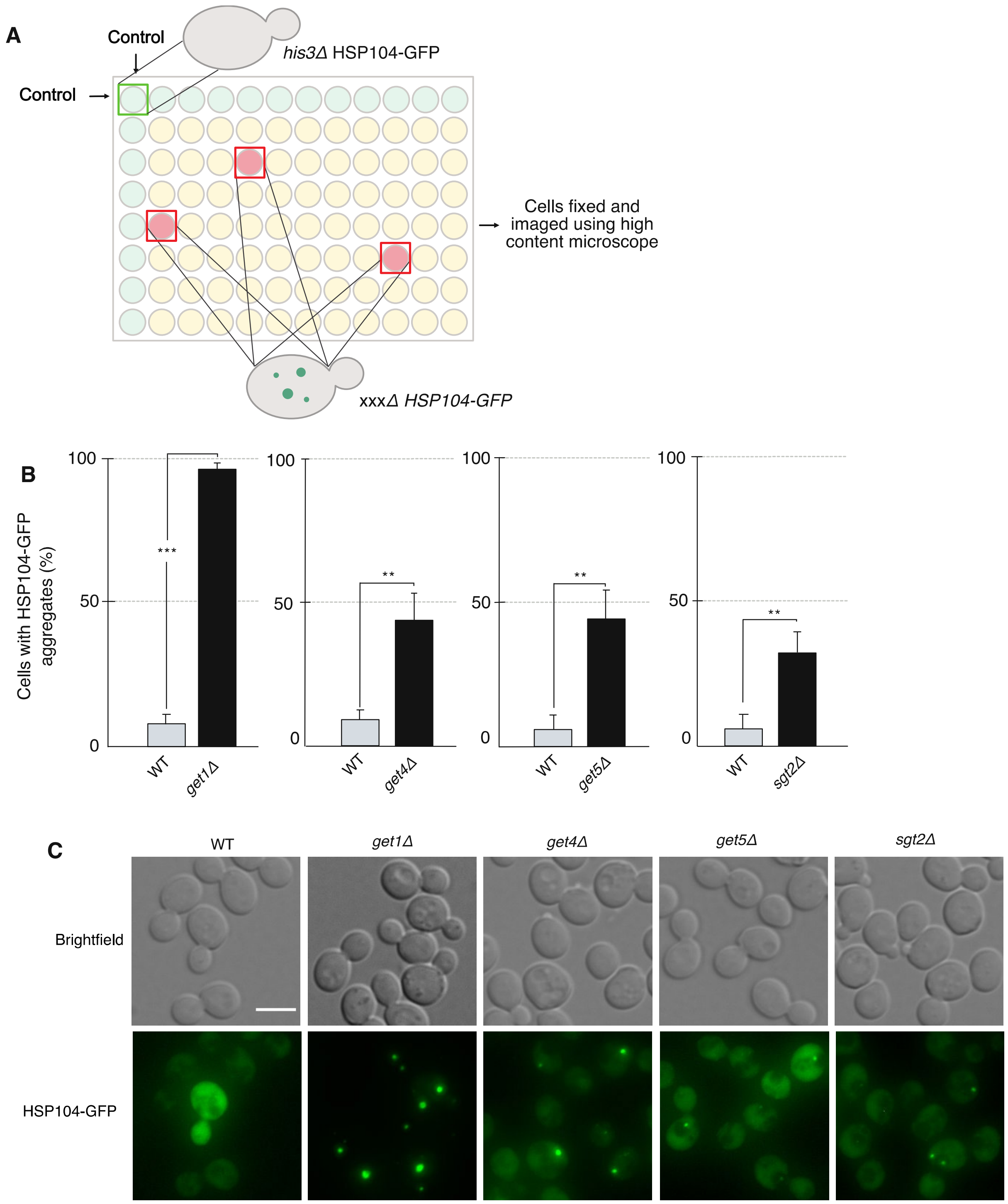

Supplementary figure S1

(**A**) Schematic image of the genome-wide screening procedure for finding genes suppressing Hsp104-GFP-associated aggregate formation in mid-exponential phase at 30°C. (**B**) Aggregate load in non-stressed wild type, *get1Δ*, *get4Δ*, *get5Δ*, and *sgt2Δ* cells growing at 30°C as determined using Hsp104-GFP. Bar graph shows the percentage of mother cells carrying Hsp104-GFP aggregates (N=3, n>200 cells per strain per replicate). (**C**)

Representative images of Hsp104-GFP aggregate load in wild type, *get1Δ*, *get4Δ*, *get5Δ*, and *sgt2Δ* cells. GFP channel displayed as maximal projection.

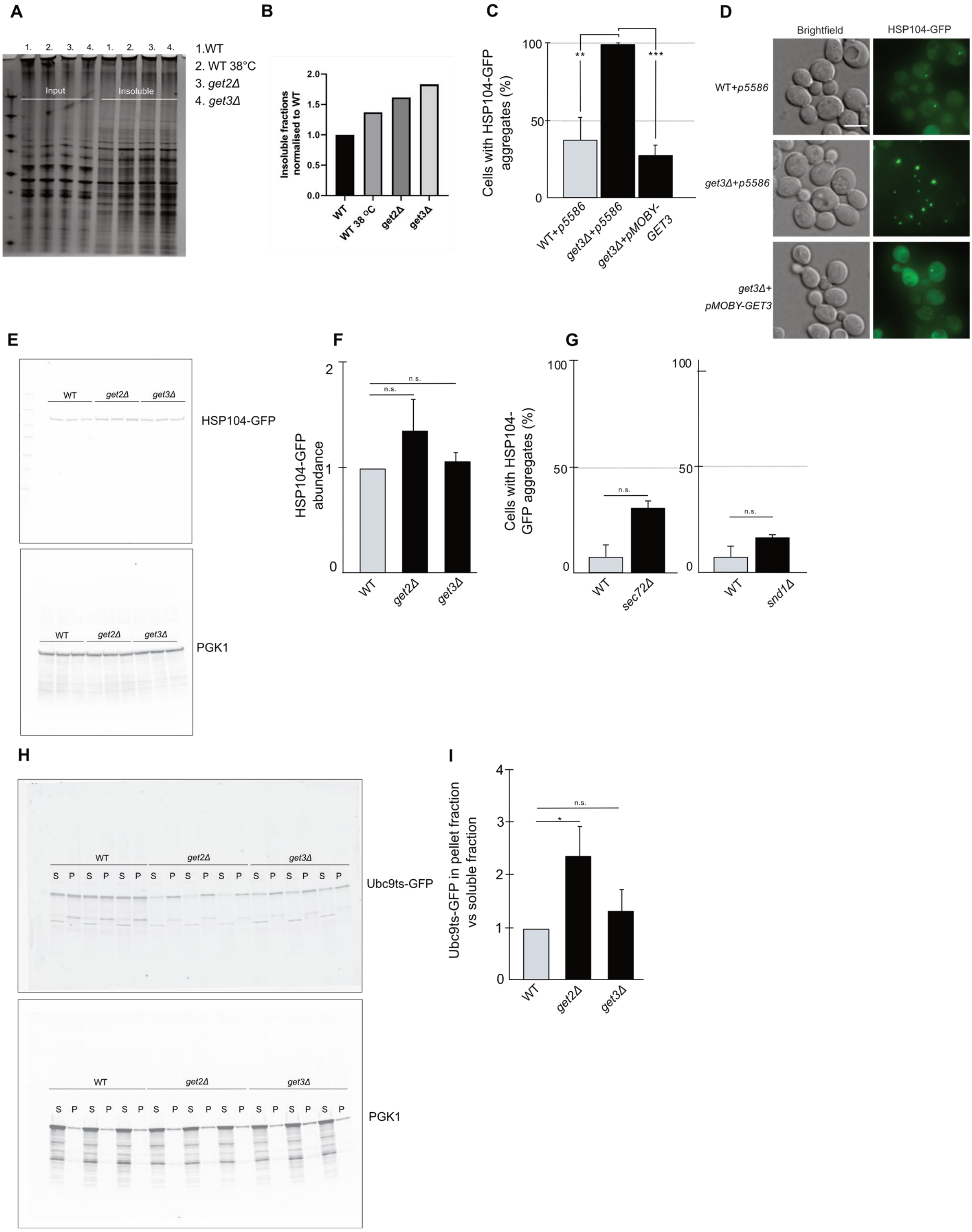

Supplementary figure S2

(**A**) Full image of silver stained cell fractions using whole cell lysates from BY4741 wild type cells, BY4741 wild type cells heat-shocked 60 min at 38°C, *get2Δ* cells and *get3Δ* cells. (**B**) Bar Graph showing the quantification of insoluble proteins fractions normalized with input and compared with WT insoluble proteins fraction. (**C**) Hsp104-GFP aggregate load in wild

type cells carrying vector control and *get3Δ* cells carrying vector control or a plasmid expressing *get3* from its endogenous promoter (midlog phase at 30°C; N=3, n>200 cells per strain per replicate). (**D**) Representative images of Hsp104-GFP aggregate load in wild type cells carrying vector control and *get3Δ* cells carrying a vector control and *get3Δ* cells complemented with the low-copy MOBY plasmid expressing *get3*. GFP channel displayed as maximal projection from 7 z slices. (**E**) Immunoblot analysis of Hsp104-GFP and Pgk1 protein levels in BY4741 wild type, *get2Δ*, and *get3Δ* cells (N=3). (**F**) Quantification of Hsp104-GFP protein levels; All samples were normalized to Pgk1 and to BY4741 Hsp104-GFP levels. (**G**) Hsp104-GFP aggregate load in *sec72Δ* and *snd1Δ* cells in midlog phase at 30°C. Bar graph shows the percentage of cells carrying Hsp104-GFP aggregates (N=3, n>200 cells per strain per replicate). GFP channel displayed as maximal projection. (**H**) Immunoblot images of Pgk1 and Ubc9ts-GFP protein levels in soluble and pellet fractions from BY4741 wild type, *get2Δ*, and *get3Δ* cells growing in mid log phase at 30°C (N=3). (**I**) Ratio of Ubc9ts-GFP in pellet and in the supernatant fraction in *get2Δ* and *get3Δ* cells compared to that of the wild type cells, which was set to a value of 1.0. Bar graphs are displayed as mean±SD. * $p<0.05$, ** $p<0.01$, *** $p<0.001$, n.s. >0.05, unpaired two-tailed t test. Scale bar 5 µm.

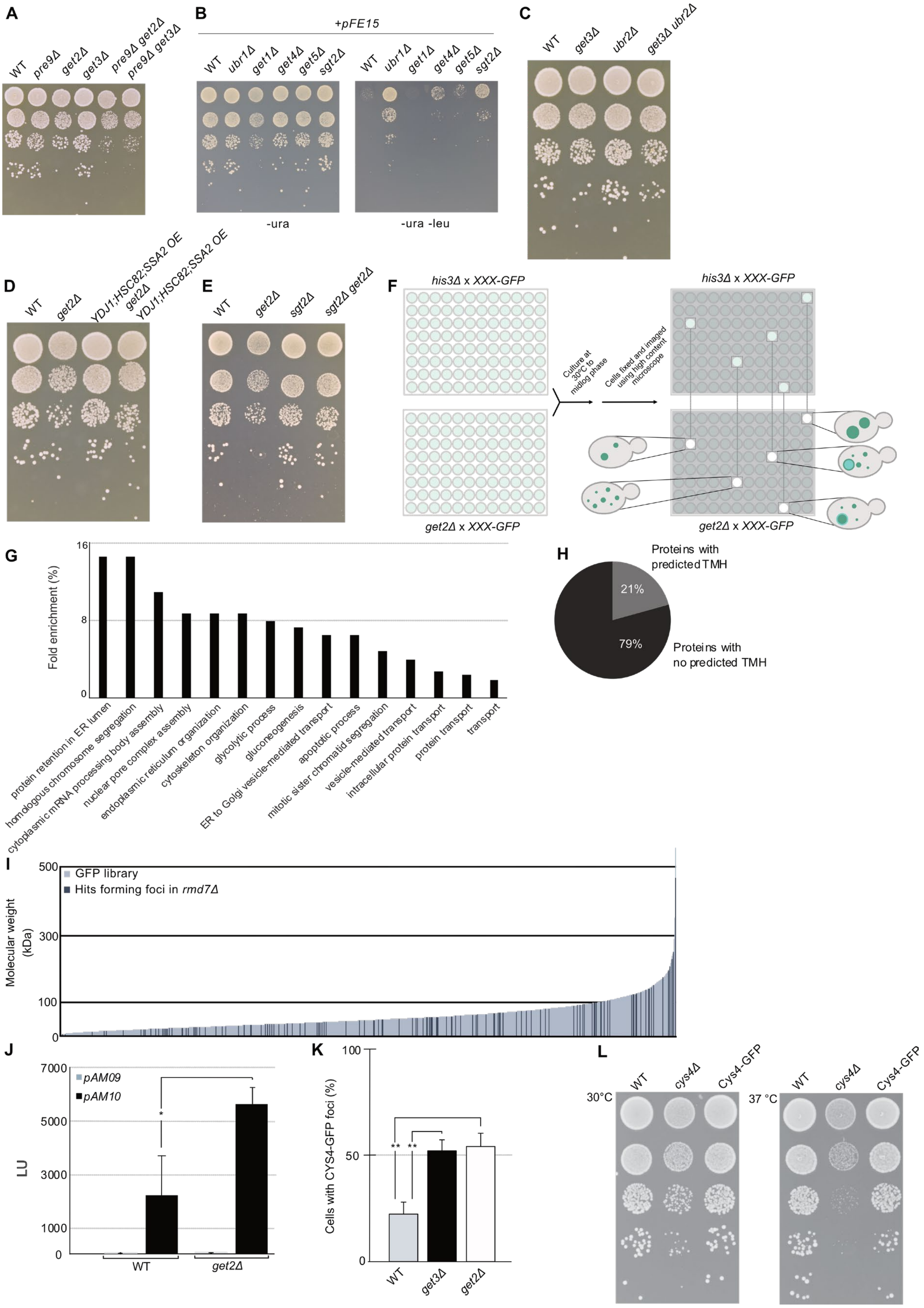
A
WT
pre9Δ
get2Δ
get3Δ
pre9Δ get2Δ
pre9Δ get3Δ
B
+pFE15
WT
ubr1Δ
get1Δ
get4Δ
get5Δ
sgt2Δ
-ura
-ura -leu
C
WT
get3Δ
ubr2Δ
get3Δ ubr2Δ
D
WT
get2Δ
YDJ1;HSC82;SSA2 OE
get2Δ YDJ1;HSC82;SSA2 OE
E
WT
get2Δ
sgt2Δ
sgt2Δ get2Δ
F
his3Δ x XXX-GFP
get2Δ x XXX-GFP
Culture at 30°C to midlog phase
Cells fixed and imaged using high content microscope
G
Fold enrichment (%)
16
8
0
protein retention in ER lumen
homologous chromosome segregation
cytoplasmic mRNA processing body assembly
nuclear pore complex assembly
endoplasmic reticulum organization
cytoskeleton organization
glycolytic process
gluconeogenesis
ER to Golgi vesicle-mediated transport
apoptotic process
mitotic sister chromatid segregation
vesicle-mediated transport
intracellular protein transport
protein transport
transport
H
Proteins with predicted TMH
21%
79%
Proteins with no predicted TMH
I
500
300
100
0
Molecular weight (kDa)
GFP library
Hits forming foci in rmd7Δ
J
7000
5000
3000
1000
0
LU
pAM09
pAM10
*
WT
get2Δ
K
100
50
0
Cells with CYS4-GFP foci (%)
**
**
WT
get3Δ
get2Δ
L
30°C
37 °C
WT
cys4Δ
Cys4-GFP

Supplementary figure S3

(**A**) Growth of BY4741 wild type, *pre9Δ*, *get2Δ*, *get3Δ, get2Δ pre9Δ* and *get3Δ pre9Δ* cells. The strains were spotted onto YPD plates in serial dilution and allowed to grow at 30°C for 2 days. (**B**) Growth of BY4741 wild type, *ubr1Δ*, *get1Δ*, *get4Δ, get5Δ* and *sgt2Δ* cells carrying ΔssCPY*-Leu2. Strains expressing ΔssCPY*-Leu2 from a plasmid (pFE15) were spotted on CSM-ura and CSM-leu-ura plates in four-fold dilution series and allowed to grow for 2 days at 30°C (N=3). (**C**) Growth of BY4741 wild type, *get3Δ*, *ubr2Δ*, *get3Δ ubr2Δ* cells. Exponentially growing cells were spotted onto YPD plates and allowed to grow for 2 days at 30°C. (**D**) Growth of BY4741 wild type, *get2Δ* cells, overexpressing *YDJ1/SSA2/HSC82* and *get2Δ* cells overexpressing *YDJ1/SSA2/HSC82.* Exponentially growing cells were spotted onto YPD plates and allowed to grow for 2 days at 30°C. (**E**) Growth of BY4741 wild type, *sgt2Δ*, *get2Δ*, *sgt2Δ get2Δ* cells. Exponentially growing cells were spotted onto YPD plates and allowed to grow for 2 days at 30°C. (**F**) A schematic figure showing the setup for screening for GFP fusions that form foci in *get2Δ* cells. (**G**) Functional enrichment of the hits from the protein aggregation screen using Database for Annotation, Visualization and Integrated Discovery (DAVID, https://david.ncifcrf.gov/). Only significantly enriched (p>0.05) GO annotations in Biological processes are presented. (**H**) Percentage of hits from the GFP foci screen with or without predicted transmembrane helices (TMH). TMH predictions were performed for hits using http://www.cbs.dtu.dk/services/TMHMM/ (now moved to https://services.healthtech.dtu.dk/service.php?TMHMM-2.0). (**I**) Distribution of proteins aggregating in the screen with respect to molecular weight in comparison to the entire GFP library. Information on molecular weight was from YeastMine. Hits YFR024C, YAR044W, YBR100W, YDR474C, YFL100W, YGL046W, YGR272C, YJL012C-A, YJL017W, YJL018W, YJL021C, YPR090W were excluded from the analysis as there is no molecular weight data for these genes in the YeastMine database. (**J**) Measurement of HSF1 activity at 30°C in wild type and *get2Δ* cells carrying a plasmid containing an Hsf1 bioluminescent reporter construct (pAM10) or the vector control plasmid (pAM09) (N=3). (**K**) CYS4-GFP foci in *get3Δ* and *get2Δ* cells. Bar graphs show the percentage of cells with ≥ 1 CYS4-GFP foci (N=3, n> 200 cells per strain per replicate). GFP channel images displayed as maximal Z projection. (**L**) Growth of BY4741 wild type, *cys4Δ* and Cys4-GFP cells. Cells were serially diluted and spotted onto YPD plates and allow to grow at 30°C and 37°C for 2 days.

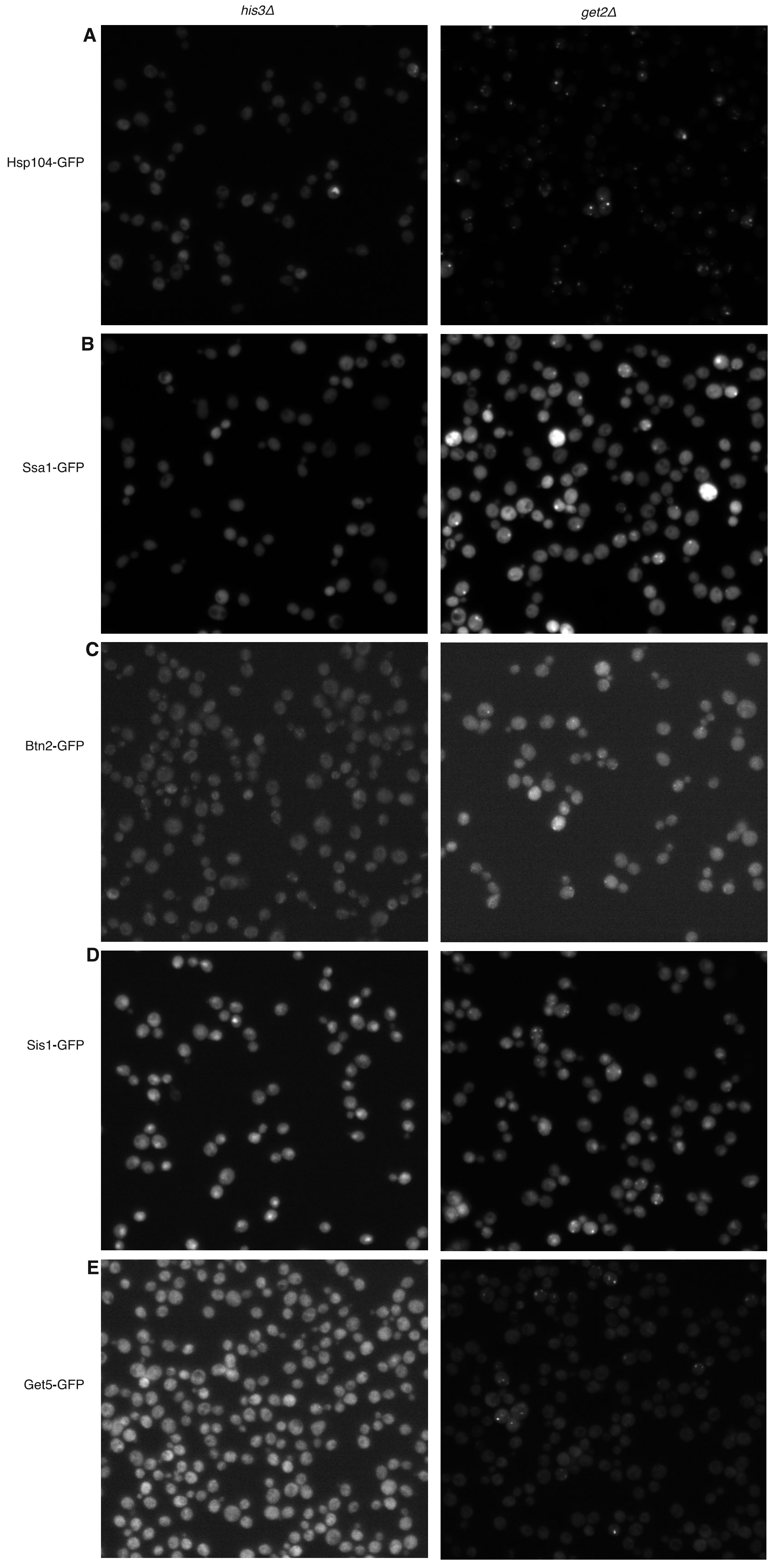
his3Δ
get2Δ
A
Hsp104-GFP
B
Ssa1-GFP
C
Btn2-GFP
D
Sis1-GFP
E
Get5-GFP

Supplementary figure S4

Images from the high content screen of the GFP-fusion strains in the *his3Δ* and *get2Δ* backgrounds, (**A**) Hsp104-GFP, (**B**) Ssa1-GFP, (**C**) Btn2-GFP, (**D**) Sis1-GFP, (**E**) Mdy2/Get5-GFP.

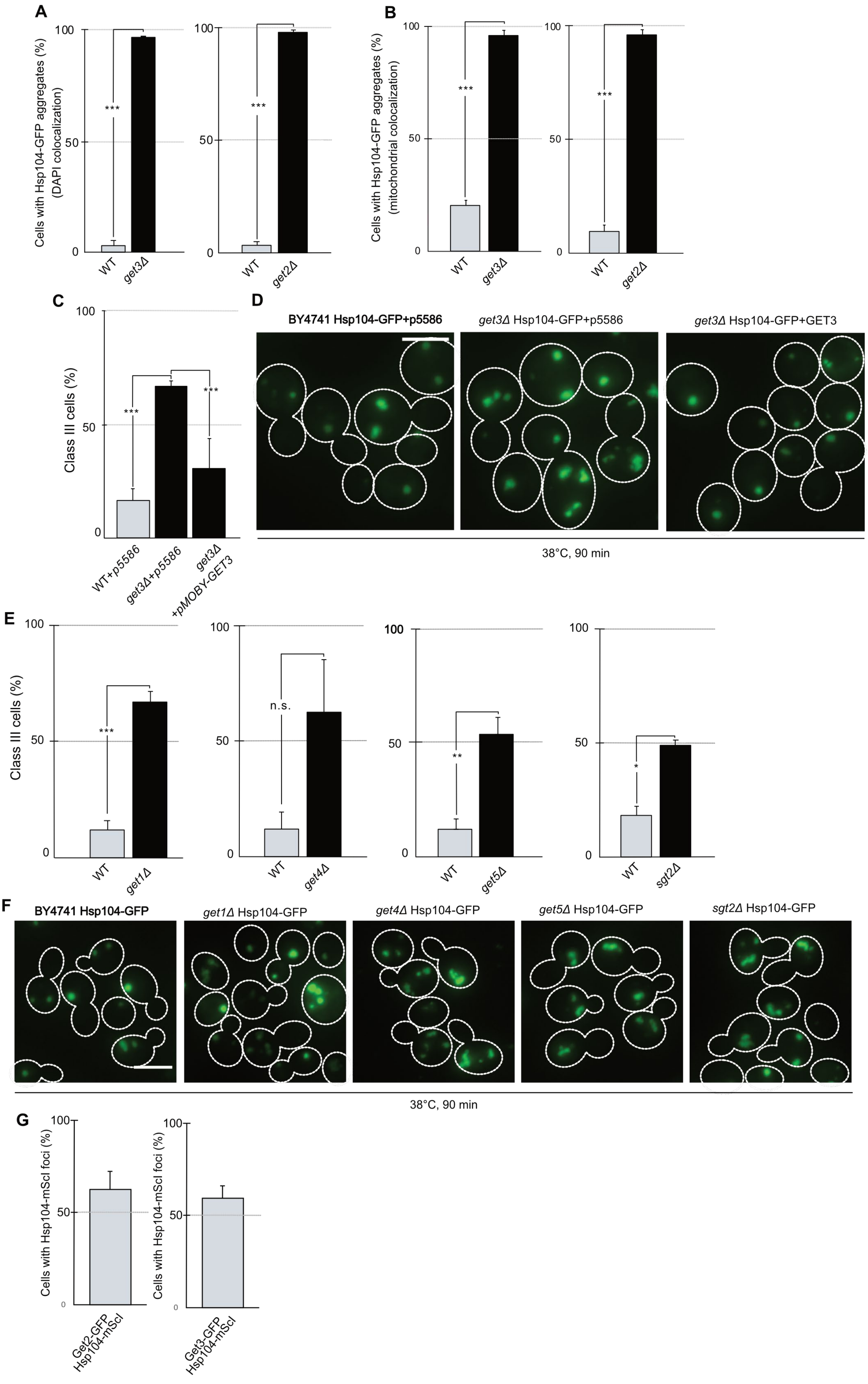
A
Cells with Hsp104-GFP aggregates (%) (DAPI colocalization)
100
50
0
***
WT
get3Δ
get2Δ
B
Cells with Hsp104-GFP aggregates (%) (mitochondrial colocalization)
C
Class III cells (%)
WT+p5586
get3Δ+p5586
get3Δ +pMOBY-GET3
D
BY4741 Hsp104-GFP+p5586
get3Δ Hsp104-GFP+p5586
get3Δ Hsp104-GFP+GET3
38°C, 90 min
E
Class III cells (%)
n.s.
**
*
get1Δ
get4Δ
get5Δ
sgt2Δ
F
BY4741 Hsp104-GFP
get1Δ Hsp104-GFP
get4Δ Hsp104-GFP
get5Δ Hsp104-GFP
sgt2Δ Hsp104-GFP
38°C, 90 min
G
Cells with Hsp104-mScl foci (%)
Get2-GFP Hsp104-mScl
Get3-GFP Hsp104-mScl

Supplementary figure S5

(A) Hsp104-GFP aggregate load in wild type, *get3Δ* and *get2Δ* cells growing in midlog phase at 30°C for studying co-localization with DAPI. Bar graph shows the percentage of cells carrying Hsp104-GFP aggregates (N=3, n>200 cells per strain per replicate). (**B**) Hsp104-GFP aggregates load in wild type, *get3Δ* and *get2Δ* cells carrying the TOM70-mRuby reporter in midlog phase at 30°C. (**C**) Failure in inclusion formation expressed as Class III cells (cells carrying three or more aggregates) in wild type cells with vector control, *get3Δ* cells carrying vector control or plasmid expressing *get3* from its endogenous promoter after a 90 min heat shock at 38°C. Bar graph shows the percentage of class III cells (N=3, n>200 cells per strain per replicate). (**D**) Representative images of Hsp104-GFP inclusion formation in *get3Δ* cells and *get3Δ* cells complemented with the low-copy MOBY plasmid expressing *get3.* GFP channel displayed as maximal projection. White outlines show mother cells and daughter cells. (**E**) Failure in inclusion formation (Class III cells) in wild type, *get1Δ*, *get4Δ*, *get5Δ*, and *sgt2Δ* cells after a 90 min heat shock at 38°C. Bar graph shows the percentage of mother cells carrying Hsp104-GFP aggregates (N=3, n>200 cells per strain per replicate). (**F**) Representative images of Hsp104-GFP inclusion formation in *get1Δ*, *get4Δ*, *get5Δ*, and *sgt2Δ* cells. GFP channel displayed as maximal projection. White outlines show mother cells and daughter cells. (G) Bar graphs shows the percentage of Hsp104-mScarletI foci aggregates in Get2-GFP and Get3-GFP cells. Bar graphs are displayed as mean±SD. * p<0.05, ** p<0.01, *** p<0.001, n.s. >0.05, unpaired two-tailed t test. Scale bar 5 µm.

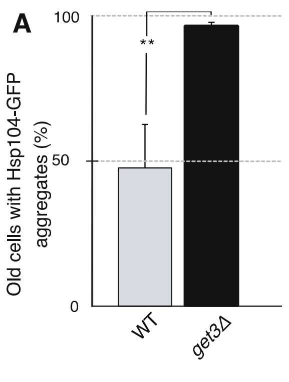

Supplementary figure S6

(**A**) Percentage of cells with Hsp104-GFP aggregates in replicatively old wild type and *get3Δ* cells isolated using magnetic bead isolation for two consecutive days (median age 12-14 generations, N=3, n>200 cells per strain per replicate).

Supplementary table S1: Hits from non-stress aggregate screen

RANK	ORF	GENE	% Cells with Aggregates	From SGD	Manually confirmed
1	YNL190W	*YNL190W*	57.37	Hydrophilin required for dessication-rehydration process	No
2	YDL100C	*GET3*	53.12	Get complex	Yes
3	YGL020C	*GET1*	41.46	Get complex	Yes
4	YER083C	*GET2*	40.93	Get complex	Yes
5	YDR378C	*LSM6*	25	Involved in mRNA decay	Yes
6	YDL020C	*RPN4*	20.01	Proteasome-related	Yes
7	YBR146W	*MRPS9*	15.39	Sunbunit of small ribosomal subunit in mitochondria	No
8	YJR032W	*CPR7*	14.64	Binds to Hsp82, contributes to chaperone acitivity	Yes
9	YGR135W	*PRE9*	13.42	20S proteasome subunit	Yes
10	YPL225W	*YPL225W*	12.61		Yes
11	YOL081W	*IRA2*	12.21	Negative Ras regulator. Required for reducing cAMP during nutrient limitation	No
12	YPL213W	*LEA1*	11.98	part of snRNP	No
13	YOR043W	*WHI2*	10.55	Required for full activation of stress response	Yes
14	YDR058C	*TGL2*	10.13	Triacylglycerol lipase in mitochondria	Yes
15	YEL004W	*YEA4*	8.63	Required for cell wall chitin synthesis	No
16	YNR072W	*HXT17*	7.6	Putatibe TM polyol transporter	No
17	YER084W	*YER084W*	7.15	Expressed at mRNA and protein level	Yes
18	YOL030W	*GAS5*	6.32	Cell wall	No
19	YPR189W	*SKI3*	6.11	RNA degradation	-
20	YDL035C	*GPR1*	5.44	Sensor integrating nutrient sensing with PKA, cAMP	-
21	YOL106W	*YOL106W*	4.85	Dubious ORF	-
22	YNL180C	*RHO5*	4.72	Involved in Pkc signaling controlling cell integrity	-
23	YOR298C-A	*MBF1*	4.6	Protein abundance increases in response to DNA replication stress	-
24	YLR452C	*SST2*	4.49	Pheromone	-
25	YDL081C	*RPP1A*	4.43	Translation regulation	-
26	YJR142W	*YJR142W*	4.35	Synthetic lethal with Pho85	-
27	YKL110C	*KTI12*	3.87	tRNA wobble nucleoside modification	-
28	YOL020W	*TAT2*	3.7	High aff tryptophan and tyrosine permease	-
29	YOR360C	*PDE2*	3.69	High aff cAMP phosphodiesterase	-
30	YPR064W	*YPR064W*	3.61	Non-essential	-
31	YNR029C	*YNR029C*	3.59		-
32	YPL224C	*MMT2*	3.54	Putative iron transporter in mitochondrial iron accumulation	-
33	YPR111W	*DBF20*	3.52	Regulates SWI5 CLB2 mRNA stability	-
34	YOR296W	*YOR296W*	3.39	Expressed during copper starvation	-

**35**	YPR201W	*ARR3*	3.35	Transports arsenite	-
**36**	YNL015W	*PBI2*	3.24	Cytosolic inhibitor of vacuolar proteinase B	-
**37**	YMR204C	*YMR204C*	3.14	Inp1. Peripheral membrane protein of peroxisomes. Involved in peroxisome inheritance	-
**38**	YPR057W	*BRR1*	3.08	snRNP	-
**39**	YPL072W	*UBP16*	2.9	DUB enzyme anchored to outer mitochondrial membrane	-
**40**	YOL063C	*MOR1*	2.85	CRT10. Induced by DNA damage	-
**41**	YMR163C	*YMR163C*	2.56		-
**42**	YNR027W	*BUD17*	2.32	Bud site selection	-
**43**	YJL213W	*YJL213W*	1.98		-
**44**	YMR174C	*PAI3*	1.91	Cytoplasmic Pep4 inhibitor	-
**45**	YMR172W	*HOT1*	1.27	Required for glycerol biosynthesis in response to osmotic stress	-

Supplementary table S2 - Hits GFP foci screen

Systematic name	Name	Brief description (from YeastMine)	Prion-like domain predicted*	Predicted intrinscal disorderedness (%)**	Number of predicted TMH***
YAL005C	SSA1	ATPase involved in protein folding and NLS-directed nuclear transport	No	26.64	0
YAL007C	ERP2	Member of the p24 family involved in ER to Golgi transport	No	22.33	2
YAL012W	CYS3	Cystathionine gamma-lyase	No	23.6	0
YAL017W	PSK1	PAS domain-containing serine/threonine protein kinase. Regulates protein synthesis and carbohydrate metabolism/storage in response to unknownfactor	No	34.51	0
YAL038W	CDC19	Pyruvate kinase. Generates pyruvate, the input for either TCA cycle or fermentation	No	26.6	0
YBL034C	STU1	Microtubule plus-end-tracking non-motor protein	No	21.41	0
YBL039C	URA7	Major CTP synthase isozyme (see also URA8)	No	22.97	0
YBL040C	ERD2	HDEL receptor	No	1.83	4
YBL063W	KIP1	Kinesin-related motor protein	Yes	26.28	0
YBL069W	AST1	Lipid raft associated protein. Interacts with ATPase Pma1 and is involved in its targeting to the plasma membrane	No	20.28	0
YBR028C	YPK3	AGC kinase. Phosphorylated by PKA in a TORC1-dependent manner, also by TORC1 directly and autophosphorylated	No	30.67	0
YBR086C	IST2	Cortical ER protein involved in ER-plasma membrane tethering	No	24.02	8
YBR126C	TPS1	Synthase subunit of trehalose-6-P synthase/phosphatase complex. Synthesizes trehalose	No	16.57	0
YCL001W	RER1	Protein involved in retention of membrane proteins	No	26.06	2
YCL040W	GLK1	Glucokinase	No	17.4	0
YDL018C	ERP3	Protein with similarity to Emp24p and Erv25p	No	25.33	1
YDL019C	OSH2	Member of an oxysterol-binding protein family with seven members. Interacts with ER anchor Scs2 at patches at plasma membrane and nuclear envelope	No	35.07	0
YDL058W	USO1	Essential protein involved in vesicle-mediated ER to Golgi transport	No	35.38	0
YDL060W	TSR1	Protein required for processing of 20S pre-rRNA in the cytoplasm	No	33.63	0
YDL072C	YET3	Protein of unknown function. Protein abundance increases with DNA replication stress	No	30.05	3
YDL099W	BUG1	Cis-golgi localized protein involved in ER to Golgi transport	No	71.26	0
YDL126C	CDC48	AAA ATPase	No	40.96	0
YDL160C	DHH1	Cytoplasmic DEAD-box helicase, stimulates mRNA decapping	No	25.69	0
YDL215C	GDH2	NAD(+)-dependent glutamate dehydrogenase	No	16%	0
YDR050C	TPI1	Triose phosphate isomerase, abundant glycolytic enzyme	No	12.1	0
YDR075W	PPH3	Catalytic subunit of protein phosphatase PP4 complex. Regulates recovery from DNA repair checkpoint	No	18.83	0
YDR111C	ALT2	Catalytically inactive alanine transaminase	No	24.26	0
YDR128W	MTC5	Subunit of SEACAT, a subcomplex of the SEA complex. SEA is a coatomer associating dynamically with vacuole. Relative distribution to vacuole membrane increases with DNA replicaiton stress	No	14.11	0
YDR141C	DOP1	Trans-Golgi network localized, leucine-zipper domain protein. Endosome to golgi transport during endocytic recycling, transport to TGN and retrograde transport TGN to Golgi	No	16.43	0
YDR233C	RTN1	Reticulon protein	No	26.44	2
YDR244W	PEX5	Peroxisomal membrane signal receptor for peroxisomal matrix proteins	No	22.71	0
YDR296W	MHR1	Mitochondrial ribosomal protein of the large subunit	No	33.63	0
YDR385W	EFT2	Elongation factor 2 (EF-2), also encoded by EFT1	No	23.63	0
YDR497C	ITR1	Myo-inositol transporter. Relative distribution to vacuole increases upon DNA replication stress	No	19.35	12
YDR502C	SAM2	S-adenosylmethionine synthetase	No	18.23	0
YDR517W	GRH1	Acetylated cis-Golgi protein, homolog of human GRASP65	No	28.61	0
YEL011W	GLC3	Glycogen branching enzyme, involved in glycogen accumulation	No	5.11	0
YEL015W	EDC3	Non-essential conserved protein with a role in mRNA decapping	No	32.67	0
YEL029C	BUD16	Putative pyridoxal kinase. Required for genome integrity, involved in bud site selection	No	12.5	0

YEL060C	PRB1	Vacuolar proteinase B (yscB) with H3 N-terminal endopeptidase activity	No	36.48	0
YEL065W	SIT1	Ferrioxamine B transporter	No	11.94	14
YER004W	FMP52	Protein of unknown function	No	2.16	0
YER077C	MRX1	Protein that associates with mitochondrial ribosome	No	22.38	0
YER111C	SWI4	DNA binding component of the SBF complex (Swi4p-Swi6p)	Yes	42.3	0
YFR024C		Deleted ORF		37.47	0
YFR049W	YMR31	Subunit of the mitochondrial alpha-ketoglutarate dehydrogenase. Mitochondrial	No	24.39	0
YGL020C	GET1	Subunit of the GET complex	No	13.19	4
YGL036W		Putative protein of unknown function	?	24.97	0
YGL037C	PNC1	Nicotinamidase that converts nicotinamide to nicotinic acid. Required for lifespan extension by caloric restriction. Responds to all known stimuli that extends life span. Protein abundance increases and cytoplasmic foci form in response to DNA replication stress	No	15.28	0
YGL140C		Putative protein of unknown function	No	21	11
YGL190C	CDC55	Regulatory subunit B of protein phosphatase 2A (PP2A)	No	23.4	0
YGL200C	EMP24	Component of the p24 complex	No	22.17	2
YGL203C	KEX1	Cell death protease essential for hypochlorite-induced apoptosis	No	29.63	1
YGR142W	BTN2	v-SNARE binding protein	No	47.56	0
YGR155W	CYS4	Cystathionine beta-synthase. Catalyzes first committed step in cysteine synthesis	No	10.26	0
YGR192C	TDH3	Glyceraldehyde-3-phosphate dehydrogenase (GAPDH), isozyme 3. Detected in cell wall and cytoplasm. Involved in glycolysis and gluconeogenesis	No	8.13	0
YGR284C	ERV29	Protein localized to COPII-coated vesicles	No	12.9	7
YHR077C	NMD2	Protein involved in the nonsense-mediated mRNA decay (NMD) pathway	No	30.17	0
YHR098C	SFB3	Component of the Sec23p-Sfb3p heterodimer of the COPII vesicle coat	No	21.96	0
YHR133C	NSG1	Protein involved in regulation of sterol biosynthesis. Forms foci in nuclear periphery in response to DNA replication stress	No	11	5
YHR159W	TDA11	Putative protein of unknown function	No	73.61	0
YHR216W	IMD2	Inosine monophosphate dehydrogenase. Catalyzes rate-limiting step in GTP biosyntheisis	No	22.37	0
YIL017C	VID28	GID Complex subunit, serves as adaptor for regulatory subunit Vid24p. In proteasome-dependent degradation of fructose-1,6-bisphosphate	No	15.64	0
YIL041W	GVP36	BAR domain protein that localizes to early and late Golgi vesicles	No	50.61	0
YIL065C	FIS1	Protein involved in mitochondrial fission and peroxisome abundance	No	23.87	1
YIL076W	SEC28	Epsilon-COP subunit of the coatomer	No	11.49	0
YIL108W		Putative metalloendopeptidase	No	15.52	0
YIR003W	AIM21	Subunit of a complex that associates with actin filaments. Increases pool of actin monomers to increases endocytic efficiency and regulates distributionofactinbetweencables and patches.	No	82.92	0
YIR004W	DJP1	Cytosolic J-domain-containing protein	No	36.81	0
YJL074C	SMC3	Subunit of the multiprotein cohesin complex	No	40	0
YJL131C	AIM23	Mitochondrial translation initiation factor 3 (IF3, mIF3)	No	43.54	0
YJL207C	LAA1	AP-1 accessory protein. Involved in TGN-endosome transport	No	10.13	0
YJR044C	VPS55	Late endosomal protein involved in late endosome to vacuole transport	No	12.86	4
YJR147W	HMS2	Protein with similarity to heat shock transcription factors	No	22.07	0
YKL060C	FBA1	Fructose 1,6-bisphosphate aldolase	No	21.45	0
YKL065C	YET1	Endoplasmic reticulum transmembrane protein	No	24.76	3
YKL073W	LHS1	Molecular chaperone of the endoplasmic reticulum lumen. HDEL protein	No	33.26	1
YKL081W	TEF4	Gamma subunit of translational elongation factor eEF1B	No	12.62	0
YKL152C	GPM1	Tetrameric phosphoglycerate mutase. 3-phosphoglycerate to 2-phosphoglycerate during glycolysis, reverse during gluconeogenesis	No	12.96	0
YKL157W	APE2	Aminopeptidase yscII	No	9.83	0
YKR013W	PRY2	Sterol binding protein involved in the export of acetylated sterols	No	52.38	0
YKR035W-A	DID2	Class E protein of the vacuolar protein-sorting (Vps) pathway	No	53.43	0
YKR054C	DYN1	Cytoplasmic heavy chain dynein	No	16.1	0
YKR078W		Cytoplasmic protein of unknown function	No	27.18	0
YLL026W	HSP104	Disaggregase	No	36.67	0

YLR083C	EMP70	Protein with a role in cellular adhesion and filamentous growth. Also endosome-to-vacuole transport	No	9.3	10
YLR127C	APC2	Subunit of the Anaphase-Promoting Complex/Cyclosome (APC/C)	No	18.29	0
YLR133W	CKI1	Choline kinase. Membrane-related	No	29.73	0
YLR257W		Unkown function	No	90.03	0
YLR264W	RPS28B	Protein component of the small (40S) ribosomal subunit	No	64.18	0
YLR330W	CHS5	Component of the exomer complex. Transport of proteins from Golgi to plasma membrane, e.g. Chitin synthase	No	63.34	0
YLR347C	KAP95	Karyopherin beta. Interacts with nucleoporins to mediate nuclear import of NLS-containing cargo proteins	No	22.3	0
YLR386W	VAC14	Enzyme regulator	No	22.39	0
YML012W	ERV25	Member of the p24 family involved in ER to Golgi transport	No	36.49	1
YML028W	TSA1	Thioredoxin peroxidase	No	18.88	1
YML081W	TDA9	Transcription factor that regulates acetate production	Yes	26.7	1
YML085C	TUB1	Alpha-tubulin	No	18.79	0
YML099C	ARG81	Zinc finger transcription factor involved in arginine-responsive genes	No	36.82	0
YML110C	COQ5	2-hexaprenyl-6-methoxy-1,4-benzoquinone methyltransferase. Mitochondrial	No	17.59	0
YML124C	TUB3	Alpha-tubulin	No	23.6	0
YMR200W	ROT1	Molecular chaperone involved in protein folding in ER	No	30.86	1
YMR300C	ADE4	Phosphoribosylpyrophosphate amidotransferase (PRPPAT). Catalyzes first step in de novo purine nucleotide biosynthesis	No	20.2	0
YNL007C	SIS1		No	39	0
YNL055C	POR1	Mitochondrial porin (voltage-dependent anion channel)	No	3.53	0
YNL074C	MLF3	Serine-rich protein of unknown function	No	59.96	0
YNL077W	APJ1	Chaperone with a role in SUMO-mediated protein degradation	No	16.48	0
YNL085W	MKT1	Protein similar to nucleases that forms a complex with Pbp1p. Formd cytoplasmic foci upon DNA replication stress	No	19.28	0
YNL126W	SPC98	Component of the microtubule-nucleating Tub4p (gamma-tubulin) complex	No	14.3	0
YNL138W	SRV2	CAP (cyclase-associated protein). Actin-related	No	29.47	0
YNL189W	SRP1	Karyopherin alpha homolog. Involved in cotranslational protein degradation. Binds ribosome-bound nascent polypeptides. Couples proteasomes to nascent polypeptides	?	36.9	0
YNL239W	LAP3	Cysteine aminopeptidase with homocysteine-thiolactonase activity. Protects against homocysteine toxicity	No	8.7	0
YNL301C	RPL18B	Ribosomal 60S subunit protein L18B	No	35.48	0
YNL312W	RFA2	Subunit of heterotrimeric Replication Protein A (RPA). SS-DNA binding protein involved in DNA repair, recombination and replication	No	23.44	0
YNR016C	ACC1	Acetyl-CoA carboxylase, biotin containing enzyme. Required for de novo synthesis of long chain fatty acids	No	29.33	0
YOL111C	MDY2	Protein involved in inserting tail-anchored proteins into ER membranes	No	24.53	0
YOR007C	SGT2	Glutamine-rich cytoplasmic cochaperone	No	50.58	0
YOR016C	ERP4	Member of the p24 family involved in ER to Golgi transport	No	15.94	1
YOR045W	TOM6	Component of the TOM (translocase of outer membrane) complex	No	22.95	1
YOR046C	DBP5	Cytoplasmic ATP-dependent RNA helicase of the DEAD-box family	No	18.26	0
YOR094W	ARF3	Glucose-repressible ADP-ribosylation factor.Regulates PIP2 levels to facilitate endocytosis	No	12.02	0
YOR285W	RDL1	Thiosulfate sulfurtransferase. Localized to mitochondrial outer membrane. Abundance increases in response to DNA replication stress	No	33.09	0
YPL061W	ALD6	Cytosolic aldehyde dehydrogenase	No	18.8	0
YPL112C	PEX25	Peripheral peroxisomal membrane peroxin	No	15.74	0
YPL119C	DBP1	Putative ATP-dependent RNA helicase of the DEAD-box protein family	No	22.37	0
YPL229W		Putative protein of unknown function	No	48.54	0
YPL242C	IQG1	Essential protein required for determination of budding pattern	No	24.48	0
YPL247C		Putative protein of unknown function	?	32.12	0
YPL274W	SAM3	High-affinity S-adenosylmethionine permease	No	19.42	11
YPR028W	YOP1	Reticulon-interacting protein	No	18.89	2
YPR035W	GLN1	Glutamine synthetase (GS). Synthesizes glutamine from glutamate and ammonia. Forms stacks of homodecamers at low pH, resulting in enzymatic inactivation. Moves from nucleus to cytoplasmic foci upon DNA replication stress	No	16.76	0
YPR097W		Protein that contains a PX domain and binds phosphoinositides	No	27.96	0
YPR129W	SCD6	Repressor of translation initiation	No	58.74	0

YPR131C	NAT3	Catalytic subunit of the NatB N-terminal acetyltransferase. Protein acetylation	No	17.95	0
YPR172W		Protein of unknown function	No	27.5	0
YPR174C		Protein of unknown function	No	57.01	0

*Prediction of prion-like domains done at http://bioinf.uab.cat/prionw/

**Disorder predicted using MobiDB. A significantly disordered protein defined as Q+N richness more than 20%, pWalz cutoff 73,55

*** Presence of transmembrane helices done using http://www.cbs.dtu.dk/services/TMHMM/

Supplementary table 3 - strains and plasmids

Strain	Genotype	Background, source	Notes
BY4741	*MATa his3Δ1 leu2Δ0 LYS2 met15Δ0 ura3Δ0*	BY4741, Euroscarf	
HSP104-GFP	*MATα can1Δ::STE2pr-Sp_his5 lyp1Δ his3Δ1 leu2Δ0 ura3Δ0 met15Δ0 HSP104-GFP::Leu2*	Y7092	Used as query straing for no stress screen
rmd7Δ	*MATα can1Δ::STE2pr-LEU2 lyp1Δ his3Δ1 leu2Δ0 ura3Δ0 LYS2+ rmd7Δ::kanmx*	Y7039	Used as query strain for GFP foci screen
BY4741 HSP104-GFP	*MATa his3Δ1 leu2Δ0 met15Δ0 ura3Δ0 HSP104-GFP-HIS3MX6*	BY4741	
BY4741 gus1-3-GFP OE	*MATa his3Δ::pRS403-pGPD-gus1-3-GFP-HIS3MX6 leu2Δ0 LYS2 met15Δ0 ura3Δ0*	BY4741	
BY4741 CYS4-GFP	*MATa his3Δ1 leu2Δ0 met15Δ0 ura3Δ0 CYS4-GFP-HIS3MX6*	BY4741	
BY4741 gus1-3-mCherry OE CYS4-GFP	*MATa his3Δ::pRS403-pGPD-gus1-3-mCherry-LEU2 leu2Δ0 LYS2 met15Δ0 ura3Δ0 CYS4-GFP-HIS3MX6*	BY4741	
arr4Δ	*MATa arr4Δ::kanmx4 his3Δ1 leu2Δ0 LYS2 met15Δ0 ura3Δ0*	BY4741	
arr4Δ	*MATa arr4Δ::natmx his3Δ1 leu2Δ0 LYS2 met15Δ0 ura3Δ0*	BY4741	
arr4Δ HSP104-GFP	*MATa arr4Δ::kanmx4 his3Δ1 leu2Δ0 met15Δ0 ura3Δ0 HSP104-GFP-HIS3MX6*	BY4741	
arr4Δ HSP104-GFP	*MATa arr4Δ::natmx4 his3Δ1 leu2Δ0 met15Δ0 ura3Δ0 HSP104-GFP-HIS3MX6*	BY4741	
arr4Δ HSP104-GFP TOM70-mRuby	*MATa arr4Δ::kanmx4 his3Δ1 leu2Δ0 met15Δ0 ura3Δ0 TOM70-mRuby-HPHMX*	BY4741	
arr4Δ CYS4-GFP	*MATa arr4Δ::kanmx4 his3Δ1 leu2Δ0 met15Δ0 ura3Δ0 CYS4-GFP-HIS3MX6*	BY4741	
arr4Δ gus1-3-GFP OE	*MATa arr4Δ::kanmx4 his3Δ::pRS403-pGPD-gus1-3-GFP-HIS3MX6 leu2Δ0 LYS2 met15Δ0 ura3Δ0*	BY4741	
ARR4-GFP HSP104-mScI	*MATa his3Δ1 leu2Δ0 met15Δ0 ura3Δ0 ARR4-GFP-HIS3MX6 HSP104-mScarlet-I-LEU2*	BY4741	
mdm39Δ	*MATa mdm39Δ::kanmx4 his3Δ1 leu2Δ0 LYS2 met15Δ0 ura3Δ0*	BY4741	
mdm39Δ HSP104-GFP	*MATa mdm39Δ::kanmx4 his3Δ1 leu2Δ0 met15Δ0 ura3Δ0 HSP104-GFP-HIS3MX6*	BY4741	
rmd7Δ	*MATa rmd7Δ::kanmx4 his3Δ1 leu2Δ0 LYS2 met15Δ0 ura3Δ0*	BY4741	
rmd7Δ	*MATa rmd7Δ::hphmx his3Δ1 leu2Δ0 LYS2 met15Δ0 ura3Δ0*	BY4741	
rmd7Δ HSP104-GFP	*MATa rmd7Δ::kanmx4 his3Δ1 leu2Δ0 met15Δ0 ura3Δ0 HSP104-GFP-HIS3MX6*	BY4741	
rmd7Δ HSP104-GFP	*MATa rmd7Δ::natmx his3Δ1 leu2Δ0 met15Δ0 ura3Δ0 HSP104-GFP-HIS3MX6*	BY4741	
rmd7Δ HSP104-GFP	*MATa rmd7Δ::hphmx his3Δ1 leu2Δ0 met15Δ0 ura3Δ0 HSP104-GFP-HIS3MX6*	BY4741	
rmd7Δ HSP104-GFP TOM70-mRuby	*MATa rmd7Δ::kanmx4 his3Δ1 leu2Δ0 met15Δ0 ura3Δ0 TOM70-mRuby-HPHMX*	BY4741	
rmd7Δ CYS4-GFP clone 1	*MATa rmd7Δ::kanmx4 his3Δ1 leu2Δ0 met15Δ0 ura3Δ0 CYS4-GFP-HIS3MX6*	BY4741	
rmd7Δ gus1-3-GFP OE	*MATa rmd7Δ::kanmx4 his3Δ::pRS403-pGPD-gus1-3-GFP-HIS3MX6 leu2Δ0 LYS2 met15Δ0 ura3Δ0*	BY4741	
RMD7-GFP HSP104-mScI	*MATa his3Δ1 leu2Δ0 met15Δ0 ura3Δ0 RMD7-GFP-HIS3MX6 HSP104-mScarlet-I-LEU2*	BY4741	
YDJ1; HSC82;SSA2 OE	MATa his3Δ1 leu2Δ0 LYS2 met15Δ0 ura3Δ0 *hsf1Δ*::PADH1YDJ1;kanMX4;PTEF1HSC82;PTDH3SSA2		
rmd7Δ YDJ1; HSC82; SSA2 OE	MATa rmd7Δ::hphmx his3Δ1 leu2Δ0 LYS2 met15Δ0 ura3Δ0 *hsf1Δ*::PADH1YDJ1;kanMX4;PTEF1HSC82;PTDH3SSA2	BY4741	
rmd7Δ YDJ1; HSC82; SSA2 OE HSP104-GFP	MATa rmd7Δ::hphmx his3Δ1 leu2Δ0 LYS2 met15Δ0 ura3Δ0 *hsf1Δ*::PADH1YDJ1;kanMX4;PTEF1HSC82;PTDH3SSA2 HSP104-GFP-HIS3MX6	BY4741	
get4Δ	*MATa get4Δ::kanmx4 his3Δ1 leu2Δ0 LYS2 met15Δ0 ura3Δ0*	BY4741	
get4Δ HSP104-GFP	*MATa get4Δ::kanmx4 his3Δ1 leu2Δ0 met15Δ0 ura3Δ0 HSP104-GFP-HIS3MX6*	BY4741	
get5Δ	*MATa get5Δ::kanmx4 his3Δ1 leu2Δ0 LYS2 met15Δ0 ura3Δ0*	BY4741	
get5Δ HSP104-GFP	*MATa get5Δ::kanmx4 his3Δ1 leu2Δ0 met15Δ0 ura3Δ0 HSP104-GFP-HIS3MX6*	BY4741	
sgt2Δ	*MATa sgt2Δ::kanmx4 his3Δ1 leu2Δ0 LYS2 met15Δ0 ura3Δ0*	BY4741	

sgt2Δ rmd7Δ HSP104-GFP	*MATa sgt2Δ::kanmx4 rmd7Δ::natmx4 his3Δ1 leu2Δ0 met15Δ0 ura3Δ0 HSP104-GFP-HIS3MX6*	BY4741	
ubr2Δ HSP104-GFP	*MATa ubr2Δ::kanmx4 his3Δ1 leu2Δ0 LYS2 met15Δ0 ura3Δ0 HSP104-GFP-HIS3MX6*	BY4741	
arr4Δ ubr2Δ HSP104-GFP	*MATa arr4Δ::natmx ubr2Δ::kanmx4 his3Δ1 leu2Δ0 LYS2 met15Δ0 ura3Δ0 HSP104-GFP-HIS3MX6*	BY4741	
pre9Δ HSP104-GFP	*MATa pre9Δ::kanmx4 his3Δ1 leu2Δ0 met15Δ0 ura3Δ0 HSP104-GFP-HIS3MX6*	BY4741	
arr4Δ pre9Δ HSP104-GFP	*MATa arr4Δ::natmx pre9Δ::kanmx4 his3Δ1 leu2Δ0 LYS2 met15Δ0 ura3Δ0 HSP104-GFP-HIS3MX6*	BY4741	
rmd7Δ pre9Δ HSP104-GFP	*MATa rmd7Δ::natmx pre9Δ::kanmx4 his3Δ1 leu2Δ0 LYS2 met15Δ0 ura3Δ0 HSP104-GFP-HIS3MX6*	BY4741	
BY4741 HSP104-GFP Nup49-mCherry	*MATa his3Δ1 leu2Δ0 met15Δ0 ura3Δ0 HSP104-GFP-HIS3MX6 nup49-mCherry-hphmx6*	BY4741	
arr4Δ HSP104-GFP Nup49-mCherry	*MATa arr4Δ::kanmx4 his3Δ1 leu2Δ0 met15Δ0 ura3Δ0 HSP104-GFP-HIS3MX6 nup49-mCherry-hphmx6*	BY4741	
rmd7Δ HSP104-GFP Nup49-mCherry	*MATa rmd7Δ::kanmx4 his3Δ1 leu2Δ0 met15Δ0 ura3Δ0 HSP104-GFP-HIS3MX6 nup49-mCherry-hphmx6*	BY4741	
**Plasmids**	**Genotype**		**Notes**
pAM09	CEN/ARS - vector control		Maser et al., 2016
pAM10	CEN/ARS - URA3 PCYC1–HSE–Nluc		Maser et al., 2016
pFE15	pRS316-PPRC1-prc1-1Δss		Eisele and Wolf, 2008
pBS35	pSB4-mCherry-hphmx6-AmpR		Addgene plasmid 83797; Hailey et al. 2002
pUbs9ts-GFP	2μ pESC: pGAL1-UBC9-ts-GFP URA3 AmpR		Kaganovich et al., 2008
p5586	MoBY: p5586-URA3-kanmx4		
pMOBY-ARR4	MoBY: ARR4-URA3-kanmx4		
pRS416	CEN - AmpR, URA3, low copy. Vector control		
pRS416-GPD	CEN - AmpR, URA3, low copy. Vector control with integrated GPD promoter		
pRS416-GPD-Aβ42	CEN - AmpR, URA3, GPD1p-Aβ42-CYC1t, low copy		Chen and Petranovic, 2015
pRS416-GPD-Htt103Q	CEN - AmpR, URA3, GPD1p-Htt103Q-GFP, low copy		Krobitsch et al., 2000
pRS316-3XHA-BirA-Bos1TMD-opsin	CEN - AmpR, URA3. pRS316-3XHA-BirA-Bos1TMD-opsin, high copy		Cho & Shan, 2018, Cho et al., 2021

Supplementary table 4 - Primers

Primer name	Purpose	5´- 3´sequence
RJ54	Upstream forward arr4	TAGTGCTGGGGATTCTCAAC
RJ55	Upstream reverse arr4	GATGGCCCAGACAAGTGTAT
RJ56	Downstream reverse mdm39	GATTGGAGACGGAGGACATT
RJ57	Upstream forward mdm39	TTGCACGTACCAACTACCTC
RJ58	Upstream forward rmd7	GGGATGAGATGGTTGCTCTT
RJ59	Downstream reverse rmd7	TGACCCATTGTGTCGTTGGA
RJ60	Upstream forward get4	GTGAGTTAGTAGCCCGTTTG
RJ61	Downstream reverse get4	GACGACAAATAGCTAGCACA
RJ62	Upstream forward get5	GCACAGGAGAACATAGTTGG
RJ63	Downstream reverse get5	TGATCGCGGTAGAGAGTATG
RJ127	Cassette switch rmd7 deltakanmx --> hphmx	CTTCCATGTTTGTAGCATCAGCAACGTAGCTCTAGGAAATACATGGAGGCCCAGAATACCCT
RJ128	Cassette switch rmd7 deltakanmx --> hphmx	TTATGAGAACAATGTATTATATTACTGAACTATCTAGAATCAGTATAGCGACCAGCATTCAC
RJ158	Confirmation rmd7 locus Upstream forward	AACTACTGGAAGCCGCAAAG
RJ159	Confirmation rmd7 locus Downstream reverse	CTAGCTCATTCTCAGGTTCC
SMH31	Amplification HSP104-GFP tag upstream forward	CCTGAGGAAGCTGAAGAATGTC
SMH35	Amplification HSP104-GFP tag downstream reverse	TTTGCTCGGGTGTCAAGTTC
SMH43	Confirmation integration kanmx region reverse	CCGTCAGCCAGTTTAGTC
SMH100	Confirmation integration mx cassette forward	CCCGTACATTTAGCCCATAC
SH9	Confirmation integration HSP104-GFP tag forward	CAATGTCGTCTTGAAGAAGGG
SH10	Confirmation integration HSP104-GFP tag reverse	ATAATGGACCAATCCGCGTG
NK433	Amplification of Nup49-mCherry::hphmx cassette forward	GTTACATCAAAAAACGAAAACACTGGCATCATTGAGCATAATGGTGAGCAAGGGCGAGG
NK434	Amplification of Nup49-mCherry::hphmx cassette reverse	GTACTTGTTATACGCACTATATAAACTTTCAGGGCGATTTACACTGGATGGCGGCGTTAGTAT
